# Strategies for Actinobacteria Isolation, Cultivation, and Metabolite Production that Are Biologically Important

**DOI:** 10.1021/acsomega.5c01344

**Published:** 2025-04-18

**Authors:** Samson Cheruiyot Koech, Michaela Plechatá, Wasu Pathom-aree, Zdenek Kamenik, Amit Jaisi

**Affiliations:** † School of Pharmacy, Walailak University, Thasala, Thai Buri, Nakhon Si Thammarat 80160, Thailand; ‡ Graduate School, Walailak University, Thasala, Thai Buri, Nakhon Si Thammarat 80160, Thailand; § Institute of Microbiology, Czech Academy of Sciences, Videnska 1083, 14200 Prague, Czech Republic; ∥ Department of Biology, Faculty of Science, Chiang Mai University, Chiang Mai 50200, Thailand; ⊥ Biomass and Oil Palm Center of Excellence, Walailak University, Thasala, Thai Buri, Nakhon Si Thammarat 80160, Thailand

## Abstract

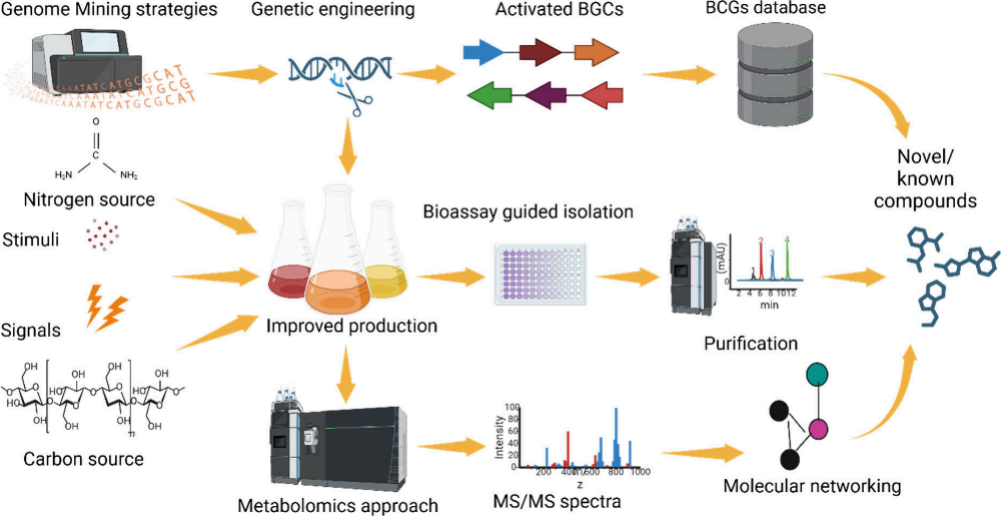

Novel antimicrobial agents are urgently needed to combat antimicrobial resistance from multidrug-resistant organisms. Actinobacteria are key sources of bioactive metabolites with diverse biological activities. Despite their contributions to drug discovery, the process from strain identification to drug manufacturing faces many challenges, especially the rediscovery of known compounds. Recent technological and scientific advancements have accelerated drug development. Efforts to isolate and screen rare actinobacterial species could yield novel bioactive compounds. This review summarizes techniques for selectively isolating rare actinobacteria, improving bioactive metabolite production, and discovering potential strains. Notably, new genomic strategies and new discoveries regarding spectroscopic signature-based bioactive natural products containing specific structural motifs are also discussed. Furthermore, this review updates the compounds derived from rare actinobacteria and their biological applications.

## 1 Introduction

**Figure 1:**
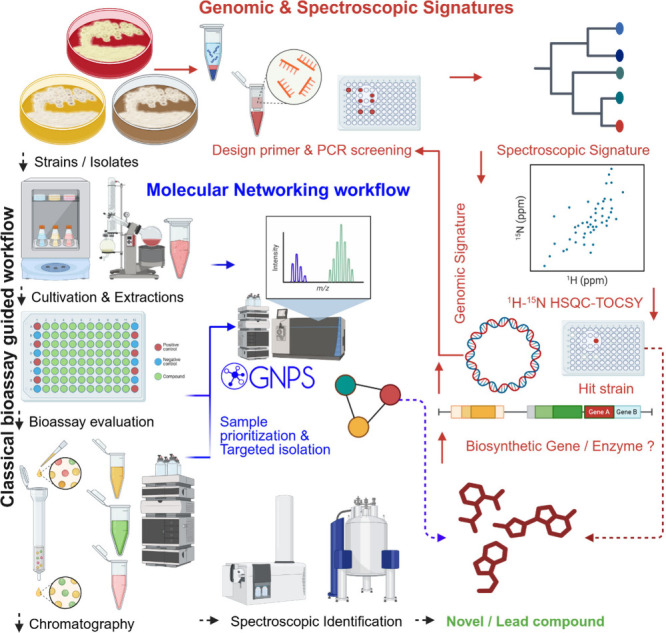
Overall process for potential strain identification, dereplication approaches, and isolation of bioactive compounds using three known approaches. Black dotted lines represent the classical approach. Blue lines represent the molecular networking approach, and red lines represent the genomic and spectroscopic approach. The colored dotted lines represent the incorporation of all approaches.

Actinobacteria are free-living microbes that can be found in a wide range of habitats, including mangroves, marine, freshwater, sediments, air, wetlands, and extreme settings including hot springs, caves, deep sea, salt lakes, deserts, and saline soil.^1,2^ Actinobacteria are Gram-positive bacteria with high guanine and cytosine (G + C) content in deoxyribonucleic acid (DNA).^3^ They are known for their ability to produce a variety of bioactive metabolites that have been utilized in the medical field, biotechnology, agriculture, and food industries.^4,5^ In addition, they are able to synthesize unique enzymes such as α-glycosidase, alkaline protease, chitinase, cellulase, and xylanase, which can be applied to the pharmaceutical industry, food industry, human medicine, and agriculture.^6,7^ The approaches for discovering bioactive compounds from actinobacteria can be broadly categorized into two groups: classical/traditional techniques and postgenomic approaches (Figure 1).^8^ In the past, the traditional drug discovery approach has been dominant, guided by bioactivity and the identification of potential candidates.^9^ In contrast, the postgenomic approach focuses on genome mining of potential isolates, hidden biosynthetic gene clusters in potential isolates, heterologous expression in other microorganisms, and bioengineering.^10,11^ Moreover, the postgenomic approach can complement and integrate with the classical approach in the discovery of bioactive metabolites.^12^

Natural compounds derived from plants, microbes, and animals have played a crucial role in drug discovery and development, contributing to 24% of all clinically used pharmaceuticals.^13^ Although it is often considered that getting novel chemicals from microbes is no longer possible due to intensive screening during the golden period, this is incorrect.^14^ Only a small percentage of bioactive metabolites have been identified; therefore, there is still opportunity for the discovery of novel compounds from microbial sources.^15^ Microbes’ potential to produce beneficial chemicals is encoded in their genome, indicating an untapped pool of natural secondary metabolites.^16^ Growing interest in the biosynthetic gene cluster component of a particular microbial species has inspired the search for bioactive compounds from microbial natural products, especially from rare actinobacteria.^17^ Additionally, the availability of cheaper sequencing technologies and genomic databases has greatly contributed to the identification of silent biosynthetic gene clusters of specific actinobacteria that are responsible for the production of secondary metabolites.^17,18^ With the recent release of Antibiotics & Secondary Metabolite Analysis Shell (ANTISMASH 7.0) (https://antismash.secondarymetabolites.org/#), a genome mining tool, it is now possible to identify, prioritize, and annotate a specific strain of actinobacteria on a genome-wide scale and dereplicate the entire process.^19^

Furthermore, high resolution mass spectrometry is considered the gold standard in profiling crude extracts both quantitatively and qualitatively. It is useful in obtaining accurate molecular mass information.^20^ Moreover, the use of online dereplication platforms such as the Global Natural Product Social Molecular Networking (GNPS)^21^ has emerged as a powerful strategy to unlock the chemical diversity of natural products and provide efficient dereplication of previously known compounds.^22^ Generally, dereplication software such as GNPS, METLIN, and natural product mass spectrometry NP-MS allows for the prioritization of unknown bioactive compounds to be isolated (Figure 1).^23^ In addition, the recent introduction of the SpecXplore interactive dashboard for mass spectral data exploration offers a better exploration of mass spectra.^24^ However, during our literature survey, data collection, and writing, several studies were published on the aspects of actinobacteria. Herein we provide for the first time the strategies for enthusiastic microbial natural product chemists with information for drug discovery approaches, identifying and updating past reviews and recent research papers (June 2022–June 2024). Notably, we have addressed the new genomic and spectroscopic signatures-based approach, whereby specific actinobacteria strains are selected after and the DNA is extracted based on specific PCR amplicons and spectroscopic signatures of genes coding for enzymes responsible for bioactive metabolites production. The active strains are also selected based on biological evaluation followed by dereplication using molecular networking (GNPS) to annotate the bioactive metabolites (Figure 1).^25^

## 2 Isolation Approaches for Rare Actinobacteria

**Figure 2:**
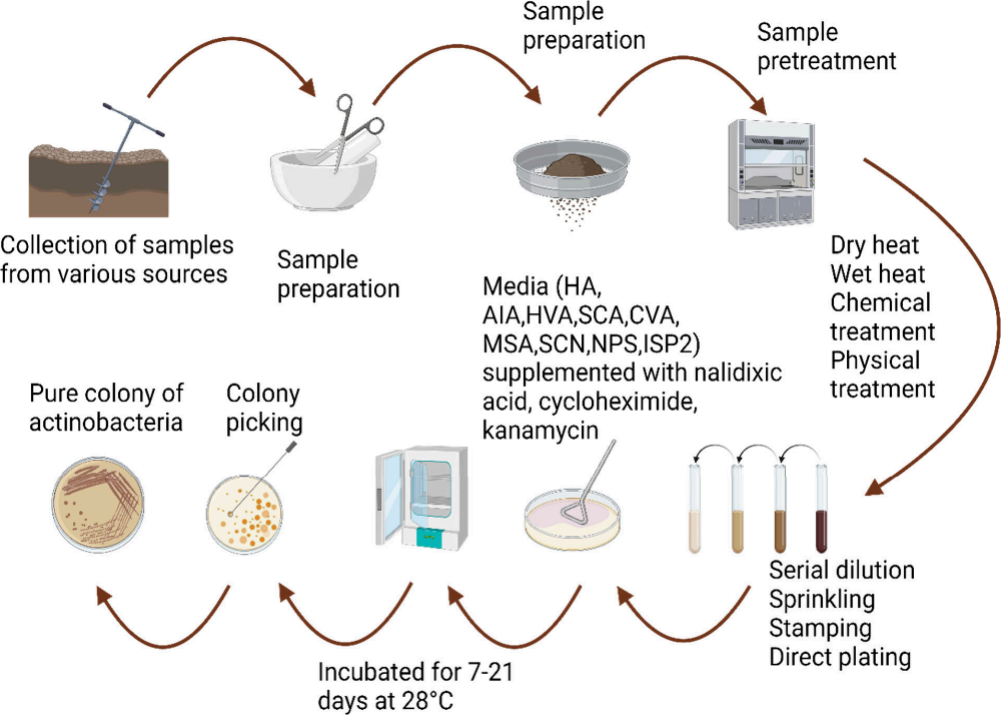
Typical steps involved in the selective isolation of actinobacteria.

The majority of the isolated actinobacteria from different habitats are mostly *Streptomyces*.^3^ It is essential to develop techniques for isolating rare actinobacterial taxa capable of synthesizing unique bioactive compounds while minimizing the reisolation of already known strains that produce specific bioactive compounds.^26,27^ Therefore, in this regard, an effective isolation approach is of paramount importance. Figure 2 summarizes the general procedures needed for the effective isolation of rare actinobacteria. Pretreatment of collected samples, different inoculation techniques, and media selection ensure the isolation of rare actinobacteria (Tables 1 and 2, Table S1).

**Table 1:** Rare Actinobacteria Selective Cultivation Media

Media	Rare Actinobacteria	Carbon Source	Nitrogen Source	Water Type	Supplements	References
Humic acid-vitamin agar (HVA)	*M. glauca*, *M. niveoalba*, *A. pusilla*, *A. roseoviolacea*, *T. mesophila*	Humic acid	Humic acid	Distilled water	B vitamins	(51)
LSV-SE agar	*M. glauca*, *M. niveoalba*, *A. pusilla*, *A. roseoviolacea*, *T. mesophila*	Lignin soil extract	Soybean flour	Distilled water	B vitamins	(51)
Humic acid vitamin gellan gum (HVG)	*Actinobispora* sp., *A. yunnanensis*	Humic acid, gellan gum	Humic acid	Not mentioned	None	(52)
Humic acid-MOPS gellan gum medium	*Rugatobispora* sp., *Actinobispora* sp., *Sporichthya* sp., *Planobispora* sp., *Planomonospora* sp.	Humic acid, gellan gum	Nitro humic acid	Distilled water	None	(53)
Marine agar (MA)	*Dermacoccus* sp., *Kocuria* sp., *Micromonospora* sp., *Tsukamurella* sp., *Williamsia* sp.	Yeast extract, peptone	Yeast extract, peptone	Not mentioned	None	(54)
Starch casein agar (SCA)	*Dermacoccus* sp., *Kocuria* sp., *Micromonospora* sp., *Tsukamurella* sp., *Williamsia* sp.	Starch casein	Casein	Not mentioned	None	(54)
Marine soil extract agar (MSA)	*Curtobacterium* sp., *Dermacoccus* sp., *Microbispora* sp., *Micromonospora* sp., *Pseudonocardia* sp., *Rhodococcus* sp., *Tsukamurella* sp.	Marine soil extract	Marine soil extract	Distilled water	None	(55)
Gause no. 2 agar (GNO2A)	*Micromonospora* sp.	Peptone, glucose	Peptone	Distilled water	None	(56)
Gause no. 2 soil extract agar (GNO2SA)	*Curtobacterium* sp., *Dermacoccus* sp., *Microbispora* sp., *Micromonospora* sp., *Pseudonocardia* sp., *Rhodococcus* sp., *Tsukamurella* sp.	Tryptone, soil extract	Peptone	Distilled water	None	(55)
Casein soil extract agar (SCSA)	*Curtobacterium* sp., *Dermacoccus* sp., *Microbispora* sp., *Micromonospora* sp., *Pseudonocardia* sp., *Rhodococcus* sp., *Tsukamurella* sp.	Casein	Casein, soil extract	Distilled water	None	(55)

**Table 2:** Approaches of Extraction of Bioactive Compounds from Rare Actinobacteria

Extraction Method	Culture Type	Solvents	Target Metabolites	Reference
Maceration	Mycelia	Ethyl acetate	Nocarpyrroline A (pyrroline derivative)	(72)
		Dichloromethane/methanol		
	Agar	Acetone	Kocurin (thiazolyl peptide)	(73)
	Agar	Ethyl acetate	Lipoxazolidinones A, B, C (oxazolidinone derivative)	(74)
Liquid–liquid extraction	Broth	Ethyl acetate	Terpenibactins A, B, C	(75)
		Methanol	Nocobactin derivatives	
	Broth	Ethyl acetate	Rifamycin W	(76)
			Protorifamycin I	
			Rifamycin W-M1 (rifamycin derivatives)	
	Broth	Ethyl acetate	1-acetyl-4-4(hydroxyphenyl)piperazine (diketopiperazine derivative)	(77)
	Broth	Ethyl acetate 1:1 methanol	Tetromadurin (polyketide polyether tetronate)	(78)
Larger adsorbents	Broth (XAD-16 resin)	Butanone	Caerulomycin A	(79)
		Acetone		
	Broth (Amberlite XAD-16N)	Methanol	Saccharothrixins D, E, F, G, I (angucycline derivatives)	(80)
	Broth (HP-20 polystyrene resin)	Methanol	Zelcovamycin (macrocyclic antibiotic)	(81)

### 2.1 High-Throughput Microbial Culturomics

The techniques involve utilizing a range of isolation media combined with various culture conditions, including specific media compositions, sample pretreatments, and adjusted incubation periods.^28^ Numerous studies, including ref (29), have reported the successful isolation of rare actinobacteria from various sources, such as the gut microbiome, using diverse isolation and pretreatment methods. Traditional isolation techniques are significantly limited in their ability to capture a large percentage of microbial diversity, despite the vast diversity of actinobacteria.^30^ In contrast, the culturomics approach proves highly effective for uncovering a wide array of actinobacteria genera and, in some cases, even identifying novel taxa.^29,31^

### 2.2 *In Situ* Methods

This approach seeks to replicate the natural conditions under which actinobacteria flourish. It involves the use of membrane chambers and diffusion reactors, combined with fruit-wrapping kraft-coated paper smeared with oligotrophic media to simulate their environment.^32^ The method has proven highly effective for isolating and recovering filamentous actinobacteria such as *Kribbella*, *Nocardioides*, and *Micromonospora*.^33^ The iChip uses miniature diffusion chambers to isolate single cells, enabling higher microbial recovery and greater taxonomic diversity than traditional agar plating, including previously uncultivated species.^34^ The iTip consists of microbeads and agar layers within the tip with its pointed end designed for placement on the surface of the target organism. The microbead layer helps to prevent disturbances from larger organisms. This technique has proven effective in isolating previously uncultivated associated bacteria.^35^

### 2.3 Flow Cell Sorting Method

Flow cell sorting is a powerful technique used for isolating and cultivating rare actinobacteria with a high successive rate.^36^ By utilizing various media commonly employed for actinobacteria isolation, the method leverages a flow cytometer to select small bacterial cells stained with SYBR Green.^37^ These cells are then sorted into 96-well plates based on their size (0.5 μm), which closely corresponds to the typical size of rare actinobacteria found in soil samples.^38^ In one study,^39^ researchers used flow cytometry to isolate single bacterial cells and filaments from marine sponges. These isolated cells underwent whole-genome amplification, allowing for the reconstruction of draft genomes. This approach led to the discovery of *Entotheonella* species, bacteria with extensive biosynthetic potential.

### 2.4 Sprinkling Technique

The sprinkling technique is an enhanced method for isolating rare actinobacteria from desert environments. Unlike serial dilution, this technique relies on a reduced moisture environment, similar to that of a desert. It is particularly effective in isolating rare actinobacteria (Figure 2).^40^ By allowing actinobacteria cells to attach directly to the culture media, this method increases the efficiency of isolating and cultivating these rare microbes such as *Georgenia*, *Microbacterium*, *Saccharopolyspora*, and *Actinomadura* species.^41^

### 2.5 Pretreatment Methods

Pretreatment of samples with heat, chemical agents, selective antimicrobial agents, and physical forces has been shown to significantly increase the isolation of rare actinobacteria, as evidenced by numerous studies (Figure 2, Table S1). Isolation of rare actinobacteria has been consistently observed in research^42−48^ after pretreatment with different methods.

### 2.6 Enrichment Method

The addition of strong chemo-attractants, such as γ-collidine, vanillin, pollen, and keratin, has proven effective in the isolation of rare actinobacteria such as *Actinoplanes*, *Catenuloplanes*, and *Dactylosporangium* species from soil samples (Figure 1, Table S1).^45−47^ The application of baiting techniques, such as pollen and keratin, has proven to be effective in isolating zoosporic actinobacteria. Additionally, a flooding solution containing skimmed milk at a pH of 9.0–10.0 stimulates *Planomonospora* spores.^48^

### 2.7 Improved Selective Isolation Media

The design of isolation media for the isolation of rare actinobacteria is of great importance to ensure the reduced growth of unwanted microbes and the recovery of rare genera of actinobacteria. Rare actinobacteria have been successfully isolated using macromolecules, such as casein, chitin, hair hydrolysate, kraft-lignin, and humic acid, as carbon and nitrogen sources.^45^ Additionally, media optimization with different carbon and nitrogen sources increases the chances of isolating novel compounds depending on target actinobacteria (Table 1, Table S1).^45,49,50^ HPLC analysis of extracts from the rare actinobacteria *Lentzea violacea* AS08 cultured in three different media, namely, CYPS (casein yeast peptone), SCP-1 (starch casein peptone), and SC (starch casein), produced different compounds. However, with starch casein media, only an additional new eudesmane sesquiterpenoids compound was purified, signifying the importance of carbon and nitrogen sources.^50^

### 2.8 Coculture

This process involves culturing two microorganisms by streaking them 2 cm apart on a solid agar plate. Subsequently, the unculturable actinobacterium is cross streaked perpendicularly. A good example is *Maribacter polysiphoniae*, which cannot grow when cultured alone but thrives in the presence of a helper strain, such as *Micrococcus luteus* (Figure 4).^57,58^ Some microbes are thought to be unable to grow independently without the presence of other microbes, as their growth depends on interspecies symbiosis through nutrient exchange (Table 3).^59,60^ Coculture techniques improve the isolation of actinobacteria by replicating their natural microbial interactions, thereby facilitating the growth of rare, slow-growing strains. Studies from the 1980s demonstrated that mixed cultures could enhance actinobacterial growth through mechanisms such as nutrient exchange, quorum sensing, and competitive stress responses.^61^ Researchers further observed that coculturing actinobacteria with fungi or other bacteria significantly increased isolation success, leading to the discovery of novel strains.^62^ Numerous custom-designed microfluidic devices have also been developed specifically to support cocultures.^63^ Community culture approaches are valuable for cultivating facultative associations and syntrophic microorganisms. When applied alongside dialysis membrane reactors, these methods have been successfully used to enrich thermophilic syntrophic anaerobic consortia.^64^

**Table 3:** Cocultured Rare Actinobacteria

Strain	Inducer	Induced Compound	Activity	References
*Nocardiopsis* sp.	*Rhodococcus wratislaviensis*	Ciromicins A and B	Cytotoxicity	(102)
*Micromonospora* sp.	*Rhodococcus* sp.	Keyicin	Antibacterial	(103)
*Micromonospora* sp.	*Tsukamurella pulmonis*	Dracolactams A and B	Antibacterial	(104)
*Catenuloplanes* sp.	*Tsukamurella pulmonis*	Catenulobactins A and B	Siderophore	(105)
*Pseudonocardiales Umezawaea* sp.	*Tsukamurella pulmonis*	Umezawamides A and B	Cytotoxicity	(106)
*Actinosynnema mirum*	*Tsukamurella pulmonis*	Mirilactams C, D, E	Unknown	(107)
*Nocardiopsis* sp.	*Actinokineospora* sp.	*N*-(2-Hydroxyphenyl)acetamide	Antibacterial and antitrypanosomal	(108)
		1,6-Dihydroxyphenazine		
		5a,6,11a,12-Tetrahydro-5a,11a-dimethyl[1,4]benzoxazino[3,2-*b*][1,4]benzoxazine		
*Rhodococcus fascians*	*Streptomyces padanus*	Rhodostreptomycins A and B	Antibacterial	(109)
*Actinokineospora spheciospongiae*	*Rhodococcus* sp.	Actinosporin E	Antimalarial	(110)
		Actinosporin G		
		Actinosporin H		
		Tetragulol		
		Capillasterquinone B		
*Saccharomonospora* sp. UR22	*Dietzia* sp. UR66	Saccharomonosporine A	Antiproliferative	(111)
		Convolutamydine F		
*Micromonospora* sp. UA17	*Gordonia* sp. UA19, *Nocardia* sp. UA23	Chlorocardicin A	Antibacterial	(112)
		Neocopiamycin A	Antifungal	
*Micromonospora* sp.	*Actinokineospora* sp.	1,6-Dicarboxylate	Antibacterial	(113)
		Phencomycin	Cytotoxic	
		Tubermycin		
		*p*-Anisamide		

## 3 Factors Influencing Isolation, Cultivation, and Strategies for Enhancing Metabolite Production from Rare Actinobacteria

The selection of carbon and nitrogen sources significantly influences the secondary metabolism in microbes.^65^ The pH affects the isolation of rare actinobacteria, especially members belonging to acidobacteria, which always require mild acidic conditions, such as *Acidobacterium capsulatum*.^66^ Adjusting the pH to a certain gradient and the addition of some calcium salts to isolation media have been found to be very effective in the isolation of rare actinobacteria obtained from Karstic caves.^67^ Temperature also plays a key role in the isolation and cultivation of rare actinobacteria; spore forming rare actinobacteria grow well at temperatures of around 28 °C.^68^ Numerous studies have demonstrated that the cultivation conditions such as solid, liquid, static, and dynamic can greatly influence the microbial metabolic process.^69^ In contrast to solid and static cultivation, dynamic cultivation in broth culture not only enables complete contact between microorganisms and nutrients but also modulates the oxygen supply and activates functional gene clusters, thereby influencing biochemical responses.^58,70^ Identification of potential strains from various sources gives a green light for the discovery of novel or known bioactive compounds that have great potential in drug discovery. However, the choice of extraction techniques, extraction solvent, and culture type greatly influences the bioactive metabolites that will be captured (Tables 1 and 2).^71^

### 3.1 Chemical Elicitation

**Figure 3:**
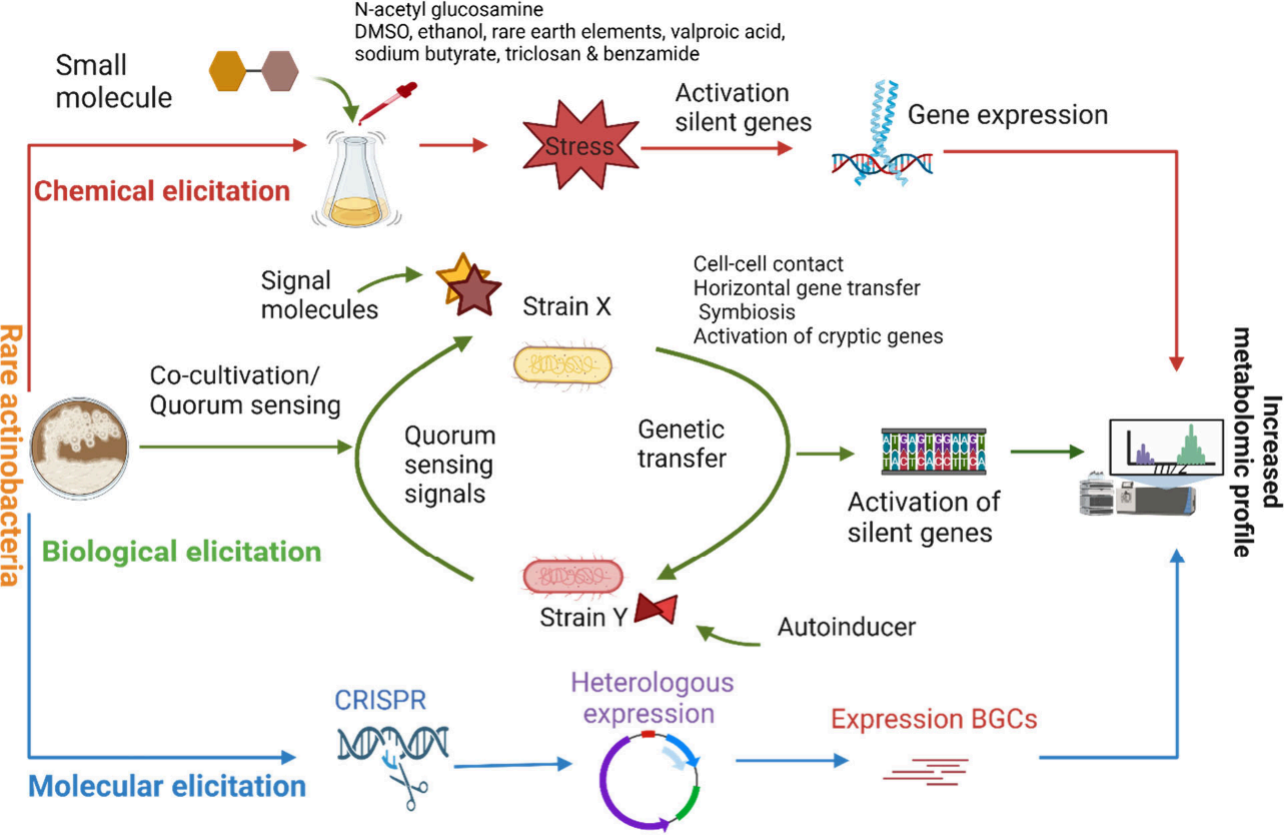
Exploitation approaches of the selected or lead bioactive compounds producing actinobacteria.

Elicitation of actinobacteria to activate silent biosynthetic gene clusters to produce bioactive metabolites is one of the strategies pursued by microbial natural product researchers.^82,83^ It has been demonstrated that physical and chemical modifications to growth media may change the rate at which some genes are transcribed and may induce biosynthetic gene cluster (BGC) expression that would normally be suppressed under laboratory culture conditions.^84^ Additions of rare earth elements and organic solvents, *N*-acetylglucosamine for example, are considered metabolic signals that can induce antibiotic synthesis and morphological differentiation in rare actinobacteria when applied directly to cell culture media. Figure 3 shows how rare actinobacteria can be elicited to increase the metabolomic profile via chemical, biological, and cocultivation approaches.^85,86^ Chemical elicitation using subinhibitory antibiotic concentrations (SICs) is a powerful strategy to activate cryptic biosynthetic pathways in rare actinobacteria. SICs influence transcription, enhancing secondary metabolite production.^87,88^ For example, triclosan and lincomycin boosted actinorhodin and salinomycin yields in *Streptomyces* species, while chloramphenicol increased actinomycin D production. Environmental stressors, such as scandium exposure, also stimulated metabolite biosynthesis. These approaches highlight SICs as effective tools for unlocking novel bioactive compounds from rare actinobacteria. Agar media enriched with radical scavengers such as SOD, catalase, ascorbic acid, and rutin significantly enhanced both the colony count and the diversity of microbial strains recovered from soil samples.^89,90^ Small molecule elicitors are essential for microbial cellular development and the activation of bioactive metabolite production. Early studies have extensively demonstrated their role in enhancing the yield of valuable bioactive compounds, particularly in *Streptomyces* species.^91−93^

### 3.2 Biological Elicitation

Cocultivation is considered a good strategy for identifying novel antimicrobial compounds from rare actinobacteria that are not produced in monocultures alone by unmasking cryptic or poorly expressed metabolites.^91,92^ Physical cell–cell interactions, the production of small molecules (such as autoregulators, quorum-sensing molecules, and siderophores), enzyme-mediated activation of metabolite precursors, and horizontal gene transfer can regulate metabolite activation or repression.^94^ It has been established that the production of bioactive metabolites by actinobacteria is influenced by mutual communication between microorganisms, and this is considered a communication signal that may hinder the production of a particular molecule while promoting the production of another.^8^ Compared with molecular and genetic engineering techniques, coculture, when fully optimized, can offer cheaper, more efficient, and simpler techniques for activating silent genes. Cocultivation allows cell-to-cell contact, which activates cryptic genes.^95,96^ The Hoshino group, which utilized a coculture of rare actinobacteria, has been successful in isolating various novel compounds, such as mirilactams, cutelobactins, and chojalactones (Figure 3 and Table 3).^98^ Cocultivation of actinobacteria with non-actinobacteria is believed to drive the structural diversity of specialized metabolites through interactions between species that coexist in the same environment.^98^ Elicitation through mixed fermentation of *Nocardia bhagyanarayanae* I-27 and the green microalga *Tetradesmus obliquus* AARL G022 resulted in the production of essential Ω-3 fatty acids, including eicosapentaenoic acid and α-linolenic acid.^99^

Quorum sensing (QS) is a key target for discovering bioactive compounds, with several QS inhibitors (QSIs) derived from microorganisms showing antimicrobial potential.^100^ Examples include ω-hydroxyrhein and emodin from *Penicillium restrictum*, which inhibit *Staphylococcus aureus* QS, and equisetin from *Fusarium* sp., which disrupts *Pseudomonas aeruginosa* biofilms. Other QSIs, such as α-pyridones from *Streptomyces* sp. and pentadecanal from *Pseudoalteromonas haloplanktis*, interfere with QS pathways, demonstrating the potential of microbial metabolites in developing novel antimicrobial agents.^101^

### 3.3 Molecular Elicitation

Mining of the microbial genomes in rare actinobacteria to identify cryptic biosynthetic gene clusters that are responsible for the synthesis of unexpressed metabolites is of immense importance and a promising area of research to discover novel bioactive compounds.^114^ CRISPR-based activation (CRISPR/Cas) is a molecular elicitation strategy for awakening silent biosynthetic gene clusters (BGCs) in actinobacteria, such as the polyketide synthase. It uses dCas9 fused to activators to enhance BGC expression, enabling the targeted activation and discovery of novel metabolites from actinobacteria.^115,116^ Current strategies include the comparison of bioactive metabolites from gene knockout mutants with the wild type of actinobacteria using bioinformatics tools, heterologous pathway expression, MALDI-TOF imaging, and *in vitro* reconstitution of the complete pathways.^117^*Amycolatopsis japonicum* was genetically manipulated by inserting a *bbr* gene encoding for the activator responsible for the biosynthesis of glycopeptide balhimycin from *Amycolatopsis balhimycina*. Results from NMR and liquid chromatography–mass spectrometry (LC-MS) showed that genetically engineered *Amycolatopsis japonicum* produced ristomycin A, which was not produced under normal laboratory conditions before (Figure 3).^118^ Transcription factor decoys are designed DNA molecules that disrupt gene regulation, potentially silencing an active biosynthetic gene cluster (BGC) or activating a previously silent one; this strategy has been applied to *Corynebacterium glutamicum*, leading to the production of bisanhydrobacterioruberin.^119^ Synthetic biology is an emerging approach for activating cryptic metabolic gene clusters in actinobacteria. This involves identifying silent biosynthetic gene clusters, amplifying them through PCR or chemical synthesis, and reconstructing them using appropriate heterologous promoters.^120^

## 4 Identification of Actinobacteria

### 4.1 Morphology and Chemotaxonomy

Microscopic morphology and chemotaxonomy are the primary criteria used to define the taxonomy of actinobacteria at the genus and species levels.^121^ The major distinguishing characteristics include the composition of the cell wall and the whole-cell sugar distribution, amino acids, lipids, and proteins, although phospholipid composition and menaquinone are also considered. Chemotaxonomy is the classification of organisms based on the distribution of their chemical components and the similarity of their cellular chemistries.^3^

### 4.2 Next Generation Sequencing

Application of whole bacterial genome sequences has increased in the last decade due to the low cost of next generation sequencing. The quality and quantity of information in the sequence data have been the most vital factors for researchers to assess.^122^ The 16S rRNA gene similarity is considered a molecular marker for prokaryotes due to its stability and universality and is highly conserved. The 16S rRNA gene similarity has proposed a cutoff point of 98.7%–99% delineation of any new species.^123^ The Eztaxon/National Center for Biotechnology Information (NCBI) servers are used for pairwise similarity, multiple sequence alignment, and construction of phylogenetic trees.^124^ Additionally, 18S rRNA and other Excloud databases such as the Ribosomal Database Project and data and tools for high-throughput rRNA analysis are also available for phylogenetic analysis.^125,126^

Actinobacteria are primarily identified using gene-specific approaches; however, as genome sequencing becomes more affordable, an increasing number of actinobacteria are categorized using genome sequencing methods.^127^ In addition, housekeeping genes are widely distributed in the population and produce proteins that are vital for metabolism.^128^ Phylogenetic relationships can be distinguished at the species level using these genes, which have a moderate evolutionary rate. Genes such as *gyr*B, *rpo*B, and *rec*A are examples of housekeeping genes.^129^ The Type Strain Genome Server is a web-based platform that allows researchers to classify prokaryotic strains based on whole-genome sequences.^130^ Average nucleotide identity compares similarities between two strains based on the whole genome of the two strains at different taxonomic levels.^131^

Metagenomics involves the identification of bioactive compounds from bacterial DNA captured from the environment and analyzing it using two approaches; it is a sequenced-based technique that employs bioinformatics tools to analyze the expression of biosynthetic gene clusters,^132^ whereas function-based metagenomics, which looks for clones that can express specific activities such as metabolite production.^133^ The two main approaches used in metagenomics includes random sequencing and targeted metagenomic sequencing.^134,135^

## 5 Approaches in the Discovery of Rare Actinobacteria with Biological Importance

**Figure 4:**
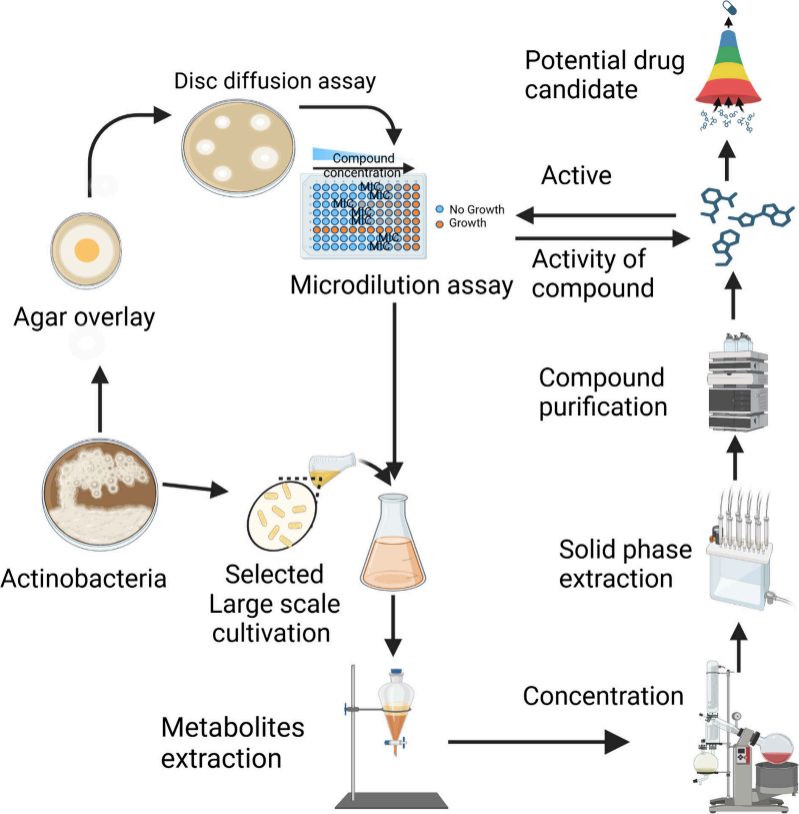
Overall process for potential strain identification and bioactive metabolites isolation.

Isolation of rare actinobacteria from various sources is a daunting task that requires a great deal of attention at every step. After isolation, the challenge is identifying the numerous numbers of actinobacteria with biological activities such as antimicrobial, antiparasitic, anticancer, and antiviral. Figure 4 shows the general identification of active strains for the biological assay and the extraction and isolation of the bioactive metabolites from potential strains.

### 5.1 Classical and High-Throughput Screening

Several studies have reported the use of both primary and secondary screenings to identify potential strains. The primary screening techniques include the streak assay method, agar plug diffusion method, agar spot method, and agar overlay method.^136^ The secondary screening bioassay techniques include the agar disk diffusion method, agar well diffusion method, and broth dilution method.^137^ To quickly identify antagonism between two microbial strains, microbiological assays such as the streak assay, agar plug diffusion method, agar spot method, and agar overlay assay are employed.^138^ To determine the antimicrobial susceptibility of isolated natural products, microbiological assays such as agar disk diffusion, agar well diffusion, and the broth dilution method are employed following the specific guidelines in each assay (Figure 4).^139^

HTS assays are effective methods for drug discovery by simultaneously examining a huge library of small molecule samples. The HTS uses 96- and 384-well plates, which allows large number of samples to be screened at once.^140^ HTS plays an important role during the early stages of drug development by qualitatively and quantitatively characterizing compound libraries necessary for preclinical and clinical absorption, distribution, metabolism, and excretion (ADME) studies. It serves to eliminate unsuitable compounds, therefore becoming cost-effective.^141^

### 5.2 Molecular Networking and Spectroscopic Approaches

Metabolomics integrates bioinformatics with high-throughput analytical techniques to enable the comprehensive identification and quantification of biological materials.^142^ Untargeted metabolomics seeks to uncover and characterize every potential cellular metabolite (often referred to as the metabolome).^143,144^ It involves the use of advanced analytical tools such as nuclear magnetic resonance (NMR) spectroscopy, high performance liquid chromatography–tandem mass spectrometry (HPLC-MS/MS), and gas chromatography–mass spectrometry (GC-MS) coupled with mass analyzers to generate data.^145^ The generated data represent a spectrum which can be putatively identified as metabolites via existing databases prior to in-depth investigation.^146^ Global Natural Product Social Molecular Networking (GNPS) enables spectral mining of larger data sets within a short time and annotation of unknown molecules.^21^

The recent introduction of the SpecXplore interactive dashboard for mass spectral data exploration offers a better exploration of mass spectra. To facilitate the exploration of local connections, SpecXplore offers two-dimensional distributed stochastic neighbor embedding.^24^ Complementary interactive visualizations include fragmentation overview maps, partial network drawings, and similarity heat maps.^24^ Instead of being based on modified cosine scores, SpecXplore is based on the pairwise similarities between the ms2deep scores. This allows it to more precisely capture the structural similarities of compounds based on their spectra via a deep-learning-based embedding representation.^147^ Another computation-based tool called Inventa was recently introduced and used in the analysis of untargeted mass spectra to discover novel compounds in natural products. Although Inventa utilizes other mass spectra analysis software, such as GNPS and MZmine, to process the data, it can combine various sets of information using bioinformatics programs, leading to the prioritization of extracts based on the ability to find novel compounds (Figure 1).^148^

Molecular networking has emerged as a powerful tool for dereplicating complex mixtures and uncovering novel natural products.^144^ Although molecular networking approaches annotate compounds based on their spectral similarities, only a small percentage of these compounds are annotated.^149^ A study utilized molecular networking to identify nocarpyrroline A, a novel bioactive compound from the strain *Nocardiopsis* sp. LX-1.^150^ However, several other computational techniques that enable metabolite annotation through *in silico* spectrum prediction and structural candidate identification have emerged to close this gap.^151^ The integration of an *in silico* database (ISDB) and molecular networking in the dereplication pipeline leads to improved annotation results for computational metabolites.^23^ Sirius utilizes the precursor *m*/*z*, isotope pattern, and MS2 spectrum to determine the molecular formula (MF) of a feature.^148^ The ZODIAC module further refines the molecular features of the annotated compound, which are subsequently utilized to identify possible structures from the database.^152^ On the other hand, the CANOPUS module performs the structural annotation of various chemical classes directly from the MS2 spectral fingerprint.^152^

Although this technique is very successful in the isolation of target compounds, it is limited largely in application to numerous microbial strains because the whole-genome sequence of the target microbial strain is required during the initial stages of identifying the potential.^153^ Genomic and spectroscopic signature assays utilize polymerase chain reaction (PCR) to screen for microbial DNA libraries, utilizing a specific set of primers designed specifically for the desired biosynthetic genes that target selective strain identification.^154^ The use of genomic and spectroscopic signatures facilitated the purification and isolation of novel compounds with diverse biological activities, including salinilactam and muanlactam from a *Micromonospora* strain.^25^ This technique focuses on the discovery of novel natural compounds with specific moieties in their structures in the absence of whole-genome data. Additionally, it allows the detection of target compounds before the purification process of the target compound commences (Figure 1).^153^

## 6 Conclusion and Future Perspectives

Antimicrobial resistance is a global threat to the healthcare system. The use of selective isolation techniques, media optimization, and enhanced techniques coupled with novel screening methodologies can lead to the discovery of rare actinobacteria that can produce novel bioactive metabolites. With the introduction of advanced sequencing techniques, genome editing tools, well-established databases for the annotation of known and unknown compounds, and the expression of silent BGCs in heterologous microbes, this combination will accelerate the discovery of new molecular structures with outstanding biological activities. However, much of this is associated with several challenges that necessitate collaboration with various platforms to effectively integrate data. This review therefore serves to guide new individuals in microbial natural product discovery by highlighting crucial factors to consider in the successive isolation of rare actinobacteria, strain prioritization, better enhanced techniques, and current advances in microbial natural products.

## Supporting Information Available

Tables with information regarding the isolation of rare actinobacteria from different environmental sources with examples of strain/family, approaches for pretreatment, and isolation media (PDF)
