# Supplementary material for: Strategies for Actinobacteria Isolation, Cultivation, and Metabolite Production that Are Biologically Important
Source: ACS Omega. 2025 Apr 18;10(16):15923–34. doi: 10.1021/acsomega.5c01344 (PMC12044489; doi:10.1021/acsomega.5c01344)
Supplement: Supplementary file 1 — ao5c01344_si_001.pdf [file ao5c01344_si_001.pdf]

SUPPLEMENTAL MATERIAL

Strategies for actinobacteria isolation, cultivation, and metabolite production that are biologically important

Samson Cheruiyot Koech[a,b*], Michaela Plechatá[c,f], Wasu Pathom-Aree[d], Zdenek Kamenik[c], Amit Jaisi[a,e]

[a]*School of Pharmacy, Walailak University, Thasala, Nakhon Si Thammarat 80160, Thailand*
[b]*Graduate School, Walailak University, Thasala, Nakhon Si Thammarat 80160, Thailand*
[c]*Institute of Microbiology, Czech Academy of Sciences, Videnska 1083, Prague, Czech Republic*
[d]*Department of Biology, Faculty of Science, Chiang Mai University, Chiang Mai 50200, Thailand*
[e]*Biomass and Oil Palm Center of Excellence, Walailak University, Thasala, Nakhon Si Thammarat 80160, Thailand*
[f]*Department of Biotechnology, University of Chemistry and Technology, Prague, Technická 5, 160 00 Prague 6-Dejvice, Czech Republic*

Correspondence Asst.Prof. Dr. Amit Jaisi, amit.ja@mail.wu.ac.th.

Abstract
Novel antimicrobial agents are urgently needed to combat the antimicrobial resistance from multidrug-resistant organisms. Actinobacteria are a key source of bioactive metabolites with diverse biological activities. Despite their contributions to drug discovery, the process from strain identification to drug manufacturing faces many challenges, especially the rediscovery of known compounds. Recent technological and scientific advancements have accelerated drug development. Efforts to isolate and screen rare actinobacterial species could yield novel bioactive compounds. This review summarizes techniques for selectively isolating rare actinobacteria, improving bioactive metabolite production, and discovering potential strains. Notably, new genomic strategy and spectroscopic signature-based new bioactive natural products containing specific structural motifs discovery are also discussed. Furthermore, this review updates the compounds derived from rare actinobacteria and their biological applications.

Keywords
Actinomycetes; Streptomyces; Antimicrobial; Dereplication; Next generation sequencing; Mass spectroscopy

List of Tables

Table S1. Isolation of rare actinobacteria from different sources

Table S1: Isolation of rare actinobacteria from different sources

Strain / Family	Pretreatment method	Isolation media (Agar)	References
**Marine**			
Isoptericola sp, *Rhodococcus* sp, *Nonomuraeae* sp, *Nocardiopsis* sp *Microbispora* sp, & *Microbacterium* sp	Washed with sterile sea water/ Incubated in acetyl trimethylammonium bromide (CTAB) buffer solution containing proteinase K	Starch-Casein-Nitrate (SCN), Raffinose-Histidine (RH), & Nutrient-Poor Sediment Extract (NPS)	1
Micromonospora sp *Rhodococcus* sp *Plantactinospora* sp *Nonomuraea* sp *Actinomadura* sp & *Streptosporangium* sp	Heated water bath 55°C	Humic acid-Vitamin (HV), SCN, Marine agar (MA), Poor Ravan saline agar (PRSA), Oligotrophic (OT), Starch-Modified Medium 1(SM1), Reasoner's 2A Agar (R2A), & Alkaliphiles Pirellula medium (M1)	2
Micromonospora sp, Saccharopolyspora sp, Actinomadura sp, Actinopolymorpha sp, Nocardiopsis sp, Saccharomonospora sp, Stackebrandtia sp & *Verrucosispora sp*	Sterile bags stored −20°C	M1, Alkaliphiles Pirellula medium (Medium 2) & Alkaliphiles Pirellula medium (Medium 3)	3
Streptomycetaceae, Pseudonocardiaceae, Nocardiopsaceae, Nocardiaceae, Promicromonosporaceae & *Micrococcaceae*	Heated 55 °C	Starch casein agar (SCA), International Streptomyces Project 2 (ISP2), Actinomycetes isolation agar (AIA), Streptomyces agar (SA), International Streptomyces Project 5 (ISP5), International Streptomyces Project 7 (ISP7), & Tap water yeast extract (TWYE)	4
Micromonospora sp, *Streptomyces* sp & *Actinomadura* sp	Dilution / Water bath 60°C	M3, NPS, & SCN	5
Micromonospora sp, *Nocardia* sp, *Actinoplanes* sp, *Nocardiopsis* sp *Saccharopolyspora* sp & *Crossiella* sp	Refrigerated 24 h / heat treatment 50°C water / irradiated by UV 254 nm / freeze dried	M1, M2, M3, M5	6
Micromonospora sp, *Nocardiopsis* sp *Rhodococcus* sp & *Saccharomonospora* sp,	Heated 120°C and incubated 55°C water bath / Shaken in a pH 7.0, 6% peptone and 0.05% SDS at 50°C / shaken and	Improved Gauze's 1, ISP2 SCA, Marine Agar 2216E (MA2216E), and M1	7

Strain / Family	Pretreatment method	Isolation media (Agar)	References
	treated with 20,000 Hz ultrasonic wave		
Nocardiopsis dassonvillei	Air dried one week / Dried 50°C	SCA	8
Nocardiopsis sp	Water bath 30°C	Starch nitrate agar (SNA)	9
Streptomycetaceae, Micromonosporaceae Gordoniaceae, Nocardiaceae, Thermomonosporaceae and *Pseudonocardiaceae*	Dried at room temperature (RT), treated with 0.05% SDS, 6% yeast extract & 1.5% phenol & washed sterilized seawater	SNA, AIA, HV, & Chitin agar	10
Microbacterium sp, *Pseudonocardia* sp, *Streptomyces* sp, *Kocuria* sp, *Aeromicrobium* sp, *Brachybacterium* sp & *Nocardiopsis* sp	Washed with sea water and kept in 25% glycerol	YE, MA2216, ISP2, M1, R2A & RH	11
Salinispora arenicola	Not mentioned (nm)	Glucose Yeast-Malt Extract Agar (GYM), & Glucose Yeast Extract Agar (GYEA)	12
Rhodococcus sp	Heated 60°C	Zobell Marine Agar (ZMA) and Poor Ravan saline (PRS)	13
Dermacoccus sp & *Micrococcus luteus*	nm	MA	14
Streptomyces sp, & *Pseudonocardia* sp	Heated 50°C water bath	R2A	15
Verrucosispora sp	nm	ISP1	16
Nocardiopsis sp	Washed with agitation in sterile seawater	SCA and Nutrient agar (NA)	17
Nocardiopsis sp	nm	GYM agar	18
Mumia sp	Glass rod homogenized with sterile seawater	Artificial seawater agar (ASA)	19
Streptomyces sp & *Rhodococcus* sp	Airdried then 65°C heated	SCA, AIA, and International Streptomyces project-Bennett's agar medium.	20
Salinispora sp, *Nocardiopsis* sp, *Verrucosispora* sp, *Micromonospora* sp, *Prauserella* sp and *Promicromonospora* sp	nm	Bennett's Rich Medium (Br medium), Mannitol Peptone Glucose Medium (MPG), ISP2, Marine Medium 1 (MM1) & Humic Acid Medium (HM)	21
Streptomyces sp, *Blastococcus* sp, *Marinactinospora* sp, *Nocardiopsis* sp, *Agromyces* sp & *Nonomuraea* sp	Sample homogenized by vigorous vertexing, exposed to	M2, M3, & HVA	22

**Strain / Family**	**Pretreatment method**	**Isolation media (Agar)**	**References**
	UV irradiation and treated with skim milk/HEPES solution		
Nocardiopsis sp	Airdried and 50° C heated	SCA	23
Actinomadura craniellae	nm	Streptomyces isolation medium.	24
Streptomycetaceae sp, *Micromonosporaceae* sp, *Nocardiaceae* sp, & *Pseudonocardiaceae* sp	Laminar flow hood dried and 55°C heated	SCN, Glycerol arginine agar (GAA) & Chitin agar (CA)	25
Nonomuraea sp	nm	ISP2, & HV	26
Actinomadura sp	nm	nm	27
Marmoricola sp	nm	nm	28
**Terrestrial soil**			
Microbacterium sp	Airdried and $CaCO_3$ treated	AIA, SCA, Glycerol Asparagine Agar (GSA), Yeast Malt Agar (YMA)/ISP2 and Bennet's agar.	29
Nonomuraea sp	4°C stored, added Ringer's solution and 60°C heated	Czapek–Dox agar (CDA)	30
Nocardiopsis sp	nm	SCA	31
Actinomadura rubrisoli	4°C stored, added Ringer's solution and 60°C heated	CDA	32
Actinokineospora sp	nm	Soil extract medium	33
Nocardiopsis sp, *Saccharopolyspora* sp, *Actinomadura* sp, *Actinocorallia* sp, *Micromonospora* sp, *Couchioplane* sp & *Planomonospora* sp	Airdried RT	SCA and HV	34
Brachybacterium sp, *Kineococcus* sp & *Microbacterium* sp	Laminar flow hood Air dried	M1-M11	35
Nocardia sp	80°C heated	ISP2 agar<br>Incubated at 28°C for 7 days	36
Nonomuraea sp	60°C heated	Medium 5336	37
Amycolatopsis sp	60°C heated	Medium 5336	38
Streptomonospora sp	nm	SCA	39
Streptomonospora litoralis	nm	SCA	40
Agromyces sp, *Kocuria* sp & *Nesterenkonia* sp	Laminar flow hood airdried RT	M1, M2 (ISP 2 medium), M3 (R2A medium), M4 (Modified Cellulose-Casein medium),	41

Strain / Family	Pretreatment method	Isolation media (Agar)	References
		M5 (CMKA medium), M6 (Raffinose-Histidine medium), M7 (Trehalose-Proline medium), M8 (Proline medium) & M10 (Casein-Glucose medium	
Amycolatopsis taiwanensis	nm	Humic-Vitamin-Yeast Medium (HVY)	42
Micromonospora sp *Actinokineospora* sp	28°C incubator air dried	GYM4 and (Zhang's Starch Soil Extract Agar) ZSSE	43
Nocardia sp	nm	AIA and (Glucose-L-Asparagine Medium) GLM	44
**Mangrove**			
Mycobacterium saopaulense	nm	ISP2	45
Streptomyces sp, *Rhodococcus* sp, *Microbacterium* sp *Micromonospora* sp, *Actinoplanes* sp & *Mycobacterium* sp	Dry heat 120 °C & phenol 1.5%	ISP 2, CDA, ISP4, NA, & Halothiobacillus HL2	46
Brevibacterium sp, *Curtobacterium* sp, *Kineococcus* sp, *Micromonospora* sp and *Mycobacterium* sp	Tween-20, NaClO, NaS_2O_3, ethanol, & $NaHCO_3$	ISP2, ISP4, Gauze No. 1, NA Halothiobacillus HL2, CDA, & ISP7	47
Nocardiopsis sp	Air dried 35°C	ISP2	48
Streptomyces sp, *Microbacterium* sp, *Agromyces* sp & *Rhodococcus* sp	Air-dried room temperature	M1-10	49
Streptomyces sp, *Curtobacterium* sp, *Mycobacterium* sp, *Micrococcus* sp, *Brevibacterium* sp, *Kocuria* sp, *Nocardioides* sp, *Kineococcus* sp, *Kytococcus* sp, *Marmoricola* sp, *Microbacterium* sp, *Micromonospora* sp *Actinoplanes* sp, *Agrococcus* sp, *Amnibacterium* sp, *Brachybacterium* sp, *Citricoccus* sp, *Dermacoccus* sp, *Glutamicibacter* sp, *Gordonia* sp, *Isoptericola* sp, *Janibacter* sp, *Leucobacter* sp, *Nocardia* sp, *Nocardiopsis* sp, *Pseudokineococcus* sp, *Sanguibacter* sp & *Verrucosispora* sp	Air dried and then ultrasonic washed	HV, ISP2, Yunnan Institute of Microbiology Medium 38 (YIM38), R2A, ISP3, RH, TWYE, GA & TP	50
Micromonospora sp	Heated 120°C, then treated with 1.5% phenol & moist heating with sterilized mangrove water	ISP1-7	51
Saccharomonospora oceani	Air-dried & $CaCO_3$ treated	HV	52

Strain / Family	Pretreatment method	Isolation media (Agar)	References
Streptomyces sp, *Nocardiopsis* sp & *Nocardioides* sp	Dried 50°C	SCA	53
Nocardiopsis sp	Dried 35°C	ISP2	48
Micromonospora sp	Air-dried	M6, M8, HV & glucose–tryptone agar medium (GP)	54
Nocardiacea sp	nm	A1 Medium Control (TM)	55
Micromonospora sp, *Actinomadura* sp, *Rhodococcus* sp, *Nocardia* sp, & *Mycobacterium* sp	Air-dried	M1, M2, M4-8	56
M. saopaulense	Tween-20, NaClO, NaS_2O_3, ethanol, & $NaHCO_3$ treated	ISP2, ISP4, Gauze No. 1, NA, Halothiobacillus HL2 & CDA	45
**Rivers**			
Microbispora sp, *Leifsonia* sp, *Verrucosispora* sp, & *Streptomyces* sp	nm	SCA	57
Nocardiopsis sp, *Saccharopolyspora* sp, *Rhodococcus* sp, *Prauserella* sp, *Amycolatopsis* sp, *Promicromonospora* sp, *Kocuria* sp, *Micrococcus* sp, *Kocuria* sp & *Micrococcus* sp	55°C heated	SCA, ISP2, AIA, SA, ISP5, ISP7 & TWYE	4
**Antarctic & arctic**			
Microbacterium sp, *Rhodococcus* sp, & *Pseudonocardia* sp	nm	A1 agar	58
Actinoplanes sp, *Arthrobacter* sp, *Kribbella* sp, *Mycobacterium* sp, *Nocardia* sp, *Pilimelia,* sp, *Pseudarthrobacter* sp, *Rhodococcus* sp, *Streptacidiphilus* sp, *Streptomyces* sp, & *Tsukamurella* sp	nm	SCA, HVA, Yeast Extract Malt Extract Agar (YEME) & Bacto-Yeast Extract	59
**Hot springs**			
Micromonospora sp and *Actinomadura* sp	Heated 50° C water bath	ISP1-6, SCA, R2A & Bushnell–Haas (BH)	60

nm; not mentioned

Starch-Casein-Nitrate agar (SCN): 10.0 g of soluble starch, 0.3 g of casein, 2 g of K_2 HPO_4, 2 g of KNO_3, 2 g of NaCl, 0.05 g of MgSO4.7H2O, 0.02 g of CaCO3, 0.01 g of FeSO4.7H2O, and 17.0 g of agar, per liter of distilled water. Raffinose-Histidine agar (RH): 10 g of raffinose, 1.0 g of L-Histidine, 1 g of K2HPO4, 0.5 g of MgSO4.7H2O, 0.01 g of FeSO4.7H2O, and 17.0 g of agar, per liter of distilled water. Nutrient-Poor Sediment Extract agar (NPS): 100 mL of marine sediment extract obtained by washing 900 mL of sediments with 500 mL of seawater and 17.0 g of agar, per liter of seawater). Humic acid-vitamin agar (HV): (Humic acid: (1.00 g); Na2 HPO4 (0.50 g); KCl (1.70 g); CaCO3 (0.02 g); FeSO4 (0.01 g); 0.00005% each of thiamine HCl, riboflavin, niacin, pyridoxine–HCl, inositol, Ca-pantothenate, p-aminobenzoic acid, and 0.000025% of biotin; Agar (18.00 g); Distilled water (1000.00 ml); pH 6.0). Marine agar (MA): Peptone 5.0g Yeast extract 1.000g Ferric citrate 0.1 g Sodium chloride 19.450

g Magnesium chloride 8.80 g Sodium sulphate 3.240g Calcium chloride 1.80 g Potassium chloride 0.550 g Sodium bicarbonate 0.160 g Potassium bromide 0.080 g Strontium chloride 0.034 g Boric acid 0.022 g Sodium silicate 0.004 g Sodium fluorate 0.003 g Ammonium nitrate 0.002 g Disodium phosphate 0.008 g. Poor Ravan saline agar (PRSA): (0.050g Peptone, 0.050 g Yeast extract 0.050 Sodium acetate 0.050 g Sodium citrate 0.050 g Pyruvic acid 0.050 g) & 15.0 g agar. Oligotrophic agar (OT): (peptone 1 g, yeast extracts 0.5 g, K2HPO4.H2O 1 g, MgSO4.7H2O 0.5 g, CaCO3 0.3 g, NaCl 5 g, vitamin mixtures, agar 15 g, pH 7.5). SM1 agar: yeast nitrogen base (67.0 g; Difco) and casamino acids (100 mg; Difco), dipotassium hydrogen phosphate (200 ml), D (-) sorbitol. SM2 agar: Yeast nitrogen base (67.0 g; Difco) and casamino acids (100 mg; Difco), dipotassium hydrogen phosphate (200 ml), D (+) melezitose. SM3 agar: Gauze's medium 2 (glucose, 10 g; peptone, 5 g; tryptone, 3 g; NaCl, 5 g; agar, 15 g; distilled water, 1 l; pH 7.0. R2A agar: contains 0.5 g of yeast extract, 0.5 g of Difco Proteose Peptone no. 3 (Difco Laboratories), 0.5 g of Casamino Acids (Difco), 0.5 g of glucose, 0.5 g of soluble starch, 0.3 g of K2HPO4, 0.05 g of MgSO4 .7H20, 0.3 g of sodium pyruvate, and 15 g of agar per liter of laboratory quality water. Adjust the pH to 7.2 with crystalline K2HPO4 or KH2PO4. M1 agar: Casein 0.3 g, Starch 10.0 g, KNO3 2.0 g, MgSO4.7H2O 0.05 g, K2HPO4 0.5 g, CaCO3 0.02 g, FeSO4.7H2O 0.01 g, Agar 20.0 g, Distilled water 1.0 L, pH 8.0. ISP 2 broth: Yeast Extract 4.0 g, Malt Extract 10.0 g, & Dextrose 4.0 g. ISP1: Pancreatic Digest of Casein 5.0 g & Yeast Extract 3.0 g. Actinomycetes isolation agar (AIA): Sodium caseinate 2.0g, L-Asparagine 0.1g, Sodium propionate 4.0g, Dipotassium phosphate 0.5g, Magnesium sulphate 0.1g, Ferrous sulphate 0.001g & Agar 15.0g. Glycerol– asparagine agar (ISP5): L-Asparagine 1.0g, Yeast extract 4.0g, Dextrose 4.0g, Calcium carbonate 2.0g, and Agar 12.0g. Tyrosine agar medium (ISP7): L-Asparagine 1.0g, L-Tyrosine 0.5g, Dipotassium hydrogen phosphate 0.5g, Magnesium sulphate heptahydrate 0.5g, Sodium chloride 0.5g, *Trace salt solution (ml) 1.0g, Agar 20.0g, *Trace salt solution contains - Ferrous sulphate heptahydrate 1.360mg Copper chloride, 2H2O 0.027mg Cobalt chloride, 6H2O, 0.040mg Sodium molybdate, dihydrate 0.025mg Zinc chloride, 0.020mg Boric acid 2.850mg Manganese chloride, tetrahydrate 1.80mg Sodium tartarate1.770mg Final pH (at 25°C) 7.3±0.1. Tap water yeast extract agar (TWYE): Peptic digest of animal tissue 5.0g Yeast extract 3.0g Agar 15.0g Final pH (at 25°C) 7.2±0.2. M2: (0.5% peptone, 0.1% yeast extract, 0.08% MgCl2, 0.6% CaCl2, 1.5% agar in filtered seawater). M3 agar: (per liter of distilled water): 0.466 g of KH2PO4, 0.732 g of Na2HPO4, 0.10 g of KNO3, 0.29 g of NaCl, 0.10 g of MgSO4.7H2O, 0.02 g of CaCO3, 200 µg of FeSO4.7H2 0, 180 µg of ZnSO4.7H2O, 15 µg of MnSO4.4H20, 4 mg of thiamine HCl (Vitamin B1) and 17 g of agar. M4: (0.5% glucose, 0.1% asparagine, 0.1% K2HPO4, 1.8% agar in filtered seawater). M5: (1.8% agar in filtered seawater) M6 (0.5% glycerol, 0.1% arginine, 0.1% K2 HPO4, 1.8% agar in filtered seawater). Improved Gauze's 1 medium: Soluble starch 20 g; KNO3, 1 g; K2HPO4·3H2O 0.5 g; MgSO4·7H2O 0.5 g; NaCl, 0.5 g; FeSO4·7H2O 0.01 g; agar, 18 g; seawater 1 L; pH 7.4–7.6 Sodium Sulfate 3.24 g, Calcium Chloride 1.8 g, Potassium Chloride 0.55 g, Sodium Bicarbonate 0.16 g, Potassium Bromide 0.08 g, Strontium Chloride 34.0 mg, Boric Acid 22.0 mg, Sodium Silicate 4.0 mg, Sodium, Fluoride 2.4 mg, Ammonium Nitrate 1.6 mg, Disodium Phosphate 8.0 mg & Agar 15.0 g. Marine agar 2216E: (Peptone 5.0 g, Yeast Extract 1.0 g, Ferric Citrate 0.1 g, Sodium Chloride 19.45 g, Magnesium Chloride 8.8 g. Trehalose dehydrates proline: Trehalose 5 g; proline 1 g; (NH4)2SO4 1 g; NaCl 1 g; CaCl2 2 g; K2HPO4 1 g; MgSO4·7H2O 1 g; Multi-vitamins: Vitamin B1, ribofavin, niacin, vitamin B6, calcium pantothenate, inositol, p-aminobenzoic acid 0.5 mg each; biotin 0.25 mg); agar 18 g; seawater 1 L; pH 7.2. Medium: starch 40.0 g; glucose 0.5 g; peptone 5.0 g; soybean powder 5.0 g; CaCO3 1.0 g; K2HPO4, 0.5 g; MgSO4, 0.5 g; agar 10.0 g; distilled water 1 L; pH 7.0–7.2). Starch nitrate agar: 20.0 g L−1 starch, 2.0 g L−1 KNO3, 1.0 g L−1 K2HPO4, 0.5 g L−1 MgSO4·7H2O, 0.5 g L−1 NaCl, 0.01 g L−1 FeSO4·7H2O, 3.0 g L−1 CaCO3, 20.0 g L−1 agar and1000 mL of 50% sea water), pH 7.2. Modified rice medium composition: 100 g commercial rice and 100 mL 50% sea water containing 0.4% yeast extract and 1% malt extract. Basal medium: 60 g NaCl, 8.36 g MgCl2·6H2O, 6.8 g MgSO4·7H2O, 0.66 g KCl, 0.5 g NH4Cl, 0.212 g C Chitin agar: Na2HPO4, 6; KH2PO4, 3; NH4Cl, 1; NaCl, 0.5; yeast extract, 0.05; agar, 15 and colloidal chitin 1% (w/v). aCl2, 15 g agar in 1liter distilled water, pH 7.5. Starch-yeast extract: 1% (w/v) starch, 0.4% (w/v) yeast extract, 0.2%, (w/v) peptone, 3.33% (w/v) artificial sea salts - Instant Ocean Brand, 1.5% (w/v) agar. Modified soil extract agar: Meat extracts 3 g, peptone 5 g,

salt mixtures*, agar 25 g, pH 7.2–7.5. YE Agar: Peptic digest of animal tissue 5.0g, Yeast extract 3.0g, & Agar 15.0g Final pH 7.2 +/- 0.2 at 25°C. GYEA (Glucose Yeast Extract Agar): Peptone 5.0g, Yeast extract 5.0g, Dextrose (Glucose) 2.0g, Potassium dihydrogen phosphate 0.5g, Dipotassium hydrogen phosphate 0.5g, Magnesium sulphate 0.3g, Sodium chloride 0.010g, Manganese sulphate 0.010g, Zinc sulphate 0.0016g, Copper sulphate 0.0016g, Copper sulphate 0.0016g, Cobalt sulphate 0.0016g & Agar 15.0g. GYM (Glucose Yeast Extract-Malt Extract Agar, DSMZ-Medium 65): Glucose 4.0 g, Yeast extract4.0 g, Malt extract 10.0 g, CaCO3 2.0 g, Agar 12.0 g and Distilled water 1000.0 ml. Poor Ravan saline: Glucose 0.05g, Peptone, 0.05g, Yeast extract 0.05g, Sodium acetate 0.05g, Sodium citrate 0.05g, Pyruvic acid 0.05g. Sponge agar: (1% macerated sponge colonies collected from the site, 50% sea water and 2.5% agar). Sea Water Agar: (50% sea water and 2.5% agar). Modified Sabouraud glucose agar: (SGA; 7.5 g casamino acid, 10.0 g yeast extract, 20.0 g MgSO4·7H2O, 3.0 g trisodium citrate·2H2O, 2.0 g KCl, 34.0 g NaCl, 10.0 µg Fe2+, 18.0 g agar, 1.0 l distilled water, pH adjusted to 7.4). Nutrient agar (NA): Peptone 5.0g, Sodium chloride 5.0g, HM peptone, 1.5g & Yeast extract 1.5g & Agar Artificial seawater agar: plate containing 0.1% pyruvate, 0.05% catechin 15.0g Final pH (at 25°C). SPY medium: with 2 mM LaCl3 (soluble starch 20 g, glucose 10 g, peptone 5 g, yeast extract 5 g, K2HPO4 0.5 g, MgSO4·7H2O 0.5 g, CaCO3 2 g, and sea salt 39.5 g, per liter). soybean meal medium: (3.3% soybean meal, 2.2% soluble starch, 2.2% glycerol, 1.2% meat extract, 2.0% peptone, and 2.2% CaCO3), starch medium (2.4% starch, 0.1% glucose, 0.5% peptone, 0.5% yeast extract, 0.3% meat extract, and 0.4% CaCO3), pharmamedia medium (1.0% pharmamedia, 0.5% glucose, 0.5% corn steep powder, 1.0% oatmeal, 0.5% K2HPO4, 0.5% MgSO4·7H2 O) and 1mL/L trace metals solution (0.1% FeSO4·7H2O, 0.1% MnCl2 ·4H2 O, 0.1% ZnSO4 ·7H2 O, 0.1% CuSO4·5H2 O and 0.1% CoCl2·6H2O) and defatted wheat germ medium (1.0% defatted wheat germ, 2.0% soluble starch, 0.5% glycerol, 0.3% meat extract, 0.3%, dry yeast, and 0.3% CaCO3). Sabouraud dextrose broth: Dextrose (Glucose) 20.0g Peptone, special 10.0g Final pH (at 25°C) 5.6±0.2. MM1: containing 6.56 g KH2PO4, 30.96 g Na2HPO4, 0.41 g MgSO4 Ã— 7 H2O, 0.088 g ferric citrate, 10 g glucose, 0.1 g L-leucine, 0.1 g L-isoleucine, 0.1 g L-valine, 0.1 g L-methionine, 0.1 g L-arginine, 0.1 g L-cysteine, 0.1 g L-glutamine, 0.5 mg riboflavin, 1 mg thiamin, 0.5 mg D-biotin, and 0.005 mg alpha -lipoic acid. M001: Peptone 5.0g, Sodium chloride 5.0g, HM peptone B# 1.5g, Yeast extract 1.5g, & Agar 15.0g. Final pH (at 25°C) 7.4±0.2. Streptomyces isolation medium: containing 3 % sea salt. Glycerol arginine agar (GAA): glycerol 10 g, asparagine 1 g, K2HPO4.H2O 1 g, MgSO4.7H2O 0.5 g, CaCO3 0.3 g, vitamin mixture of HV medium 3.7 mg, and agar 15 g, pH 7.2. A-16 production medium: consisting of glucose 2%, Pharmamedia (Trader's Protein) 1%, CaCO3 0.5%, Corporation, 50 g l−1). Bn-2 agar medium: (soluble starch 0.5%, glucose 0.5%, meat extract (Kyokuto Pharmaceutical Industrial Co., Ltd.) 0.1%, yeast extract (Difco Laboratories) 0.1%, NZ-case (Wako Chemicals USA, Inc.) 0.2%, NaCl 0.2%, CaCO3 0.1%, and agar 1.5% in distilled water of pH 7.0). A11M production medium: (glucose 0.2%, soluble starch 2.5%, yeast extract 0.5%, polypeptone (Wako Pure Chemical Industries, Ltd.) 0.5%, NZ-amine (Wako Pure Chemical Industries, Ltd.) 0.5%, CaCO3 0.5%, and Diaion® HP-20 1% in distilled water of pH 7.0). Basal medium: 60 g NaCl, 8.36 g MgCl2·6H2O, 6.8 g MgSO4·7H2O, 0.66 g KCl, 0.5 g NH4Cl, 0.212 g CaCl2, 15 g agar in 1liter distilled water, pH 7.5. Czapek–Dox agar: Sucrose 20 g, NaNO3 2 g, K2HPO4 1 g, MgSO4.7H2O 0.5 g, KCl 0.5 g, FeSO4.7H2O 0.01 g, vitamin mixtures 3.7 mg, agar 25 g, pH 7.2. Soil extract medium: Meat extracts 3 g, peptone 5 g, salt mixtures*, agar 25 g, pH 7.2–7.5. TSB (trypticase soy broth): Pancreatic digest of casein: 1.7g, Papaic digest of soybean meal: 0.3g, Sodium chloride (NaCl): 0. 5g, Dibasic potassium phosphate (K2HPO4): 0.25g, Glucose monohydrate: 0.25g in 100 ml distilled water pH after sterilization (at 25°C): 7.3±0.2. M6 media: Raffinose 5.0 g, Histidine 1.0 g, KNO3 1.0 g, NaCl 1.0 g, CaCl2 2.0 g, K2HPO4 1.0 g, MgSO4 .7H2O 1.0 g, Trace salt 1.0 mL, Agar 20.0 g, Distilled water 1.0 L, pH 8.0. M7 media: Trehalose 5.0 g, L-Proline 1.0 g, (NH4)2SO4 1.0 g, CaCl2 2.0 g, NaCl 1.0 g, K2HPO4 1.0 g, MgSO4.7H2O 1.0 g, Vitamin mixture 1.0 mL, Agar 20.0 g, Distilled water 1.0 L, pH 8.0. M8: Glucose 1.0 g, peptone 0.5 g, tryptone 0.3 g, NaCl 30.5 g, Vitamin mixture 1.0 mL, Agar 20.0 g, Distilled water 1.0 L, pH 8.0. M9 media: Glucose 1.0 g, peptone 0.5 g, tryptone 0.3 g, NaCl 0.5 g, Vitamin mixture 1.0 mL, Agar 20.0 g, Distilled water 1.0 L, pH 8.0. M10: R2A 18.6 g (BD), NaCl 30.0 g, Agar 12.0 g, H2O 1.0 L, pH 8.0. M11 media: Chitin 2.0 g, K2HPO4 0.7 g, KH2 PO4 0.3 g, MgSO4.7H2O 0.5 g, FeSO4.7H2O

0.01 g, $ZnSO_4$,0.001g, $MnCl_2$ 0.001g, Agar 12.0 g, Distilled water 1.0 L, pH 8.0. Medium 5336: (soluble starch (10 g/L), casein (peptone Typ M) (1 g/L), K_2HPO_4 (0.5 g/L), $MgSO_4$. $7H_2O$ (5.0 g/L), and agar (20 g/L). Medium: composed of 15 g of glucose, 15 g of soybean meal, 5 g of corn steep liquor, 2 g of $CaCO_3$ and 5 g of NaCl in 1 L distilled water, pH was adjusted to 7.0. Medium 5294: (1% soluble starch, 0.2% yeast extract, 1% glucose, 1% glycerol, 0.25% corn steep liquor, 0.2% peptone, 0.1% NaCl, 0.3% $CaCO_3$; pH 7.2 GYM4 media: Composed of 4 g each of glucose, yeast extract and malt extract per litre of demineralized water. ZSSE agar media: Containing starch and soil extract. GLM agar media: Yeast extract, 3 g; malt extract, 3 g; peptone Type I, 5 g; starch, 10 g; agar, 20 g; distilled water, 1000 mL. ISP 4: Soluble Starch 10g/L, Dipotassium Phosphate 1g/L, Magnesium Sulfate USP 1g/L, Sodium Chloride 1g/L, Ammonium Sulfate 2g/L, Calcium Carbonate 2g/L, Ferrous Sulfate 1mg/L, Manganous Chloride 1mg/L, Zinc Sulfate 1mg/L, Agar 20g/L. Gauze No. 1: (soluble starch, 20.0 g; ferrous sulfate, 0.01 g; sodium chloride, 0.5 g; potassium nitrate, 1.0 g; magnesium sulfate, 0.5 g; dipotassium hydrogen phosphate, 0.5 g; agar, 15.0 g; distilled water 1 L, pH 7.2). Nutrient agar: Peptone, 10.0 g; sodium chloride, 5.0 g; beef extract, 3.0 g; agar, 15.0 g; distilled water 1 L, pH 7.2). Halothiobacillus HL2 medium: Glucose, 10.0 g; tryptone, 3.0 g; peptone, 5.0 g; NaCl, 5.0 g; agar, 20.0 g; distilled water 1 L, pH 7.2). Czapek agar. ISP 7: L-Asparagine 1.0g L-Tyrosine 0.5g Dipotassium hydrogen phosphate 0.5g Magnesium sulphate heptahydrate 0.5g Sodium chloride 0.5g *Trace salt solution (ml) 1.0g Agar 20.0g. YIM 38 broth medium: yeast extracts 4 g; glucose 4 g; malt extracts 10 g; thiamine–HCl, riboflavin, niacin, pyridoxin-HCl, inositol, calcium pentothenate, p-aminobenzoic acid, each 0.5 mg, and biotin 0.25 mg; pH 7.2. TWYE: Yeast extract (0.25 g); K_2HPO_4 (0.50 g); Agar (18.0 g); Tap water (1000.00 ml). TP agar: Trehalose (5.0 g); proline (1.0 g); $(NH_4)_2SO_4$ (1.0 g); NaCl (1.0 g); $CaCl_2$ (2.00 g); K_2HPO_4 (1.0 g); $MgSO_4.7H_2O$ (1.00 g); Agar (20.0 g); vitamin mixture (1.00 ml), Distilled water (1000.00 ml); pH 7.2. CM: Microcrystalline cellulose (10.0 g); Casein (0.30 g); KNO_3 (0.20 g); K_2 HPO_4 (0.50 g); $CaCO_3$ (0.02 g); $FeSO_4$ (0.01 g); NaCl (100 g); $MgCl_2.6H_2O$ (30 g), KCl (20 g); Agar (15.0 g); Distilled water (1000.0 ml); pH 7.5. GA agar: Soluble starch (20.0 g); KNO_3 (1.0 g); K_2HPO_4 (0.50 g); $MgSO_4.7H_2O$ (0.05 g); $FeSO_4{\cdot}7H_2O$ (0.01 g); Agar (20.0 g); Distilled water (1000.0 ml); pH 7.4-7.6. GP (glucose–tryptone agar medium): Casein enzymic hydrolysate, 5.0g, Yeast extract, 3.0g, Glucose, 1.0g & Agar, 15.0g in 1sliter deionized water. A1 Medium: Control (TM), where the sediment was amended with 1 L of A1 medium (1 L mangrove filtered water, 2 g peptone, 10 g starch, 4 g yeast extract. Modified 2216 medium broth: soybean cake (20 g), maltodextrin (10 g), peptone (5.0 g), yeast extract (10 g), glucose (10 g), NaCl (19.45 g), $MgCl_2{\cdot}6H_2O$ (12.6 g), $MgSO_4{\cdot}7H_2O$ (6.64 g), $CaCl_2$ (1.8 g), KCl (8.0 mg), $SrCl_2{\cdot}6H_2O$ (57 mg), and ferric citrate (0.1 g). SG broth: Glucose 20.0 g, yeast extract 5.0 g, Soytone 10 g, $CoCl_2.6H_2O$ (1.0 mg) and calcium carbonate 2.0 g in 1 liter of demineralized water. The suspension (pH 7.2). GLM medium: Yeast extract, 3.0g, malt extract 3.0g, peptone 5.0g, glucose: 10.0g (pH- 7.2). CYSP medium: Casein hydrolysate 10.0g, starch 10.0g, yeast extract 1.0g, peptone 1.0g, (pH- 7.2). MGYP medium: Maltose 3.0g, glucose: 10.0g, yeast extract 3.0, peptone 5.0g, (pH- 7.2). Medium 333: Glucose 5.0g, peptone 3.0g, soluble starch 10.0g, yeast extract 3.0g, $CaCO_3$ 2.0g, NH_4NO_3 3.0g, (pH- 7.2). A 1 agar: starch, 10 g l^{-1}; yeast extract, 4 g l^{-1}; peptone, 2 g l^{-1}; agar, 14 g l^{-1}), SW agar: (agar, 14 g l^{-1}). SC agar: starch, 10 g l^{-1}; KNO_3, 2 g l^{-1}; K_2 HPO_4, 2 g l^{-1}; casein, 0.3 g l^{-1}; $MgSO_4$ $\cdot 7H_2O$, 0.05 g l^{-1}; $CaCO_3$, 0.02 g l^{-1}; $FeSO_4$ $\cdot 7H_2O$, 0.01 g l^{-1}; agar, 18 g l^{-1}. Yeast Extract Malt Extract Agar (YEME): Bacto-Yeast Extract (Difco) 4.0g, Bacto-Malt Extract (Difco) 10.0 g, Bacto-Dextrose (Difco) 4.0 g, & i Bacto agar 20.0g in 1000 ml Distilled water.

References

1. Girão, M.; Ribeiro, I.; Ribeiro, T.; Azevedo, I. C.; Pereira, F.; Urbatzka, R.; Leão, P. N.; Carvalho, M. F. Actinobacteria Isolated From *Laminaria ochroleuca*: A Source of New Bioactive Compounds. *Front. Microbiol.* 2019, 10. https://doi.org/10.3389/fmicb.2019.00683
2. Veyisoglu, A.; Tatar, D. Diversity and antimicrobial activity of culturable actinobacteria isolated from the sediment of Sarıkum Lake. *Biotechnology & Biotechnological Equipment*. 2021. 35(1), 1136-1146. https://doi.org/10.1080/13102818.2021.1952898
3. Pinto-Almeida, A.; Bauermeister, A.; Luppino, L.; Grilo, I.R.; Oliveira, J.; Sousa, J.R.; Petras, D.; Rodrigues, C.F.; Prieto-Davó, A.; Tasdemir, D.; et al. The Diversity, Metabolomics Profiling, and the Pharmacological Potential of Actinomycetes Isolated from the Estremadura Spur Pockmarks (Portugal). *Mar. drugs*. 2021. 20(1), 21. https://doi.org/10.3390/md20010021
4. Zothanpuia; Passari, A. K.; Leo, V. V.; Chandra, P.; Kumar, B.; Nayak, C.; Hashem, A.; Abd Allah, E. F.; Alqarawi, A. A.; Singh, B. P. Bioprospection of actinobacteria derived from freshwater sediments for their potential to produce antimicrobial compounds. *Microb. Cell Factories*. 2018. 17(1), 68. http://europepmhttps://doi.org/10.1186/s12934-018-0912-0
5. Ribeiro, I.; Girão, M.; Alexandrino, D.A.M.; Ribeiro, T.; Santos, C.; Pereira, F.; Mucha, A.P.; Urbatzka, R.; Leão, P.N.; Carvalho, M.F. Diversity and Bioactive Potential of Actinobacteria Isolated from a Coastal Marine Sediment in Northern Portugal. *Microorganisms.* 2020. 8(11). https://doi.org/10.3390/microorganisms8111691
6. Gozari, M.; Bahador, N.; Jassbi, A. R.; Mortazavi, M. S.; Hamzehei, S.; Eftekhar, E. Isolation, distribution and evaluation of cytotoxic and antioxidant activity of cultivable actinobacteria from the Oman Sea sediments. *Acta Oceanol Sin*. 2019. 38(12), 84-90. https://doi.org/10.1007/s13131-019-1515-2
7. Chen, L.; Wang, Z.; Du, S.; Wang, G. Antimicrobial Activity and Functional Genes of Actinobacteria from Coastal Wetland. *Curr Microbiol.* 2021. 78(8), 3058-3067. https://doi.org/10.1007/s00284-021-02560-3
8. Dhanaraj, S.; Thirunavukkarasu, S.; Allen John, H.; Pandian, S.; Salmen, S.H.; Chinnathambi, A.; Alharbi, S.A. Novel marine *Nocardiopsis dassonvillei*-DS013 mediated silver nanoparticles characterization and its bactericidal potential against clinical isolates. Saudi J. Biol. Sci. 2020. 27(3), 991-995. https://doi.org/https://doi.org/10.1016/j.sjbs.2020.01.003
9. Hamed, A.; Abdel-Razek, A.S.; Frese, M.; Stammler, H.G.; El-Haddad, A.F.; Ibrahim, T.M.A.; Sewald, N.; Shaaban, M. Terretonin N: A New Meroterpenoid from *Nocardiopsis* sp. *Molecules*. 2018. 23(2). https://doi.org/10.3390/molecules23020299
10. Sangkanu, S.; Rukachaisirikul, V.; Suriyachadkun, C.; Phongpaichit, S. Antifungal activity of marine- derived actinomycetes against *Talaromyces marneffei*. *J. Appl. Microbiol*. 2021, 130(5), 1508-1522. https://doi.org/10.1111/jam.14877
11. Liu, T.; Wu, S.; Zhang, R.; Wang, D.; Chen, J.; Zhao, J. Diversity and antimicrobial potential of Actinobacteria isolated from diverse marine sponges along the Beibu Gulf of the South China Sea. *FEMS Microbiol. Ecol.* 2019, 95(7). https://doi.org/10.1093/femsec/fiz089
12. Contreras-Castro, L.; MartÍNez-GarcÍA, S.; Cancino-Diaz, J. C.; Maldonado, L. A.; HernÁNdez-Guerrero, C. J.; MartÍNez-DÍAz, S. F.; GonzÁLez-Acosta, B.; Quintana, E. T. Marine Sediment Recovered *Salinispora sp.* Inhibits the Growth of Emerging Bacterial Pathogens and other Multi-Drug-Resistant Bacteria. *Pol J Microbiol.* 2020, 69(3), 321-330. https://doi.org/10.33073/pjm-2020-035
13. Baig, U.; Dahanukar, N.; Shintre, N.; Holkar, K.; Pund, A.; Lele, U.; Gujarathi, T.; Patel, K.; Jakati, A.; Singh, R.; et al. Phylogenetic diversity and activity screening of cultivable Actinobacteria isolated from marine sponges and associated environments from the western coast of India. *Access Microbiol.* 2021, 3(9), 000242. https://doi.org/10.1099/acmi.0.000242

14. Santos, J. D.; Vitorino, I.; De la Cruz, M.; Díaz, C.; Cautain, B.; Annang, F.; Pérez-Moreno, G.; Gonzalez Martinez, I.; Tormo, J. R.; Martín, J. M.; et al. Bioactivities and Extract Dereplication of Actinomycetales Isolated From Marine Sponges. *Front. Microbiol*. 2019, 10. 727. https://doi.org/10.3389/fmicb.2019.00727
15. Flores Clavo, R.; Ruiz Quiñones, N.; Hernández-Tasco, Á.J.; José Salvador, M.;Tasca Gois Ruiz, A.L.; Fantinatti, F. Evaluation of antimicrobial and antiproliferative activities of Actinobacteria isolated from the saline lagoons of northwestern Peru. *PloS one.* 2021, 16(9), https://doi.org/10.1371/journal.pone.0240946
16. Nair, V.; Kim, M.C.; Golen, J.A.; Rheingold, A.L.; Castro, G.A.; Jensen, P.R.; Fenical, W. Verrucosamide, a Cytotoxic 1,4-Thiazepane-Containing Thiodepsipeptide from a Marine-Derived Actinomycete. *Marin. Drugs*. 2020, 18(11), 549. https://doi.org/10.3390/md18110549
17. Matroodi, S.; Siitonen, V.; Baral, B.; Yamada, K.; Akhgari, A.; Metsä-Ketelä, M. Genotyping-Guided Discovery of Persiamycin A From Sponge-Associated Halophilic *Streptomonospora* sp. PA3. *Front. Microbiol*. 202, 11, 1237-1237. https://doi.org/10.3389/fmicb.2020.01237
18. Xu, D.; Nepal, K. K.; Chen, J.; Harmody, D.; Zhu, H.; McCarthy, P. J.; Wright, A. E.; Wang, G. Nocardiopsistins A-C: New angucyclines with anti-MRSA activity isolated from a marine sponge-derived *Nocardiopsis sp*. HB-J378. *Synth. Syst. Biotechnol*. 2018, 3(4), 246-251. https://doi.org/10.1016/j.synbio.2018.10.008
19. Kimura, T.; Tajima, A.; Inahashi, Y.; Iwatsuki, M.; Kasai, H.; Mokudai, T.; Niwano, Y.; Shiomi, K.; Takahashi, Y.; Ōmura, S.; et al. Mumiamicin: Structure and bioactivity of a new furan fatty acid from *Mumia* sp. YSP-2-79. *J. Gen. Appl. Microbiol.* 2018, 64(2), 62-67. https://doi.org/10.2323/jgam.2017.06.004
20. Pavan Kumar, J.; Gomathi, A.; Gothandam, K.M.; Vasconcelos, V. Bioactivity Assessment of Indian Origin-Mangrove Actinobacteria against *Candida albicans*. *Mar. Drugs*. 2018, 16(2). https://doi: 10.3390/md16020060
21. Yang, N.; Song, F. Bioprospecting of Novel and Bioactive Compounds from Marine Actinomycetes Isolated from South China Sea Sediments. *Curr. Microbiol.* 2018, 75(2), 142-149. https://doi.org/10.1007/s00284-017-1358-z
22. Ng, Z.Y.; Tan, G.Y. Selective isolation and characterisation of novel members of the family Nocardiopsaceae and other actinobacteria from a marine sediment of Tioman Island. *Antonie Van Leeuwenhoek*. 2018. 111(5), 727-742. https://doi.org/10.1007/s10482-018-1042-8
23. Siddharth, S.; Rai, V.R. Isolation and characterization of bioactive compounds with antibacterial, antioxidant and enzyme inhibitory activities from marine-derived rare actinobacteria, *Nocardiopsis* sp. SCA21. *Microb. Pathog*. 2019, 137, 103775. https://doi:10.1016/j.micpath.2019.103775
24. Li, F.; Liu, S.; Lu, Q.; Zheng, H.; Osterman, I. A.; Lukyanov, D. A.; Sergiev, P. V.; Dontsova, O. A.; Liu, S.; Ye, J.; et al. Studies on Antibacterial Activity and Diversity of Cultivable Actinobacteria Isolated from Mangrove Soil in Futian and Maoweihai of China. *J Evid Based Complementary Altern Med.* 2019, 3476567-3476567. https://doi.org/10.1155/2019/3476567
25. Gozari, M.; Zaheri, A.; Jahromi, S.T.; Gozari, M.; Karimzadeh, R. Screening and characterization of marine actinomycetes from the northern Oman Sea sediments for cytotoxic and antimicrobial activity. *Int Microbiol.* 2019, 22(4), 521-530. https://doi.org/10.1007/s10123-019-00083-3
26. Yang, T.; Yamada, K.; Zhou, T.; Harunari, E.; Igarashi, Y.; Terahara, T.; Kobayashi, T.; Imada, C. Akazamicin, a cytotoxic aromatic polyketide from marine-derived *Nonomuraea* sp. *J. Antibiot*. 2019, 72(4), 202-209. https://doi.org/10.1038/s41429-018-0139-7
27. Zhang, Z.; Zhou, T.; Yang, T.; Fukaya, K.; Harunari, E.; Saito, S.; Yamada, K.; Imada, C.; Urabe, D.; Igarashi, Y. Nomimicins B-D, new tetronate-class polyketides from a marine-derived actinomycete of the genus *Actinomadura*. *J. Org. Chem.* 2021, 17, 2194-2202. https://doi.org/10.3762/bjoc.17.141

28. Tan, Y.; Shan, Y.; Zheng, R.; Liu, R.; Sun, C. Characterization of a Deep-Sea Actinobacterium Strain Uncovers Its Prominent Capability of Utilizing Taurine and Polyvinyl Alcohol. *Frontiers in Microbiology*. 2022. 13. https://doi.org/10.3389/fmicb.2022.868728
29. Bano, N.; Siddiqui, S.; Amir, M.; Zia, Q.; Banawas, S.; Iqbal, Roohi. D. Bioprospecting of the novel isolate *Microbacterium proteolyticum* LA2(R) from the rhizosphere of *Rauwolfia serpentina*. *Saudi Journal of Biological Sciences*. 2022, 29(3), 1858-1868. https://doi.org/10.1016/j.sjbs.2021.10.038
30. Ay, H. *Nonomuraea terrae* sp. nov., isolated from arid soil. *Arch Microbiol*. 2020, 202 (8), 2197-2205. https://doi.org/10.1007/s00203-020-01941-9
31. Messaoudi, O.; Sudarman, E.; Bendahou, M.; Jansen, R.; Stadler, M.; Wink, J. Kenalactams A-E, Polyene. Macrolactams Isolated from *Nocardiopsis* CG3. *J. Nat. Prod.* 2019, 82(5), 1081-1088. https://doi.org/10.1021/acs.jnatprod.8b00708
32. Ay, H. Genomic insight into a novel actinobacterium, *Actinomadura rubrisoli* sp. nov., reveals high potential for bioactive metabolites. *Antonie Van Leeuwenhoek*. 2021, 114(2), 195-208. https://doi.org/10.1007/s10482-020-01511-5
33. Wang, Y.; Shi, J.; Liu, T.; Zhang, Y.; Zhang, L.; Zhang, X. *Actinokineospora xionganensis* sp. nov., a filamentous actinomycete isolated from the lakeside soil of Baiyangdian. *Antonie Van Leeuwenhoek*. 2021, 114(5), 487-496. https://doi.org/10.1007/s10482-021-01532-8
34. Messaoudi, O.; Wink, J.; Bendahou, M. Diversity of Actinobacteria Isolated from Date Palms Rhizosphere and Saline Environments: Isolation, Identification and Biological Activity Evaluation. *Microorganisms*. 2020, 8(12), 1853. https://doi.org/10.3390/microorganisms8121853
35. Wang, Y.; Shi, J.; Liu, T.; Zhang, Y.; Zhang, L.; Zhang, X. *Actinokineospora xionganensis* sp. nov., a filamentous actinomycete isolated from the lakeside soil of Baiyangdian. *Antonie Van Leeuwenhoek.* 2021, 114(5), 487-496. https://doi.org/10.1007/s10482-021-01532-8
36. Zhang, L.; Zhang, J.; Ren, B.; Lu, W.; Hou, C.; Wang, J.; Ma, X.; Ma, R.; Liu, M.; Liu, Z.; et al. Characterization of anti-BCG benz[α]anthraquinones and new siderophores from a Xinjiang desert-isolated rare actinomycete *Nocardia sp.* XJ31. *Appl. Microbiol. Biotechnol*. 2020, 104(19), 8267-8278. https://doi.org/10.1007/s00253-020-10842-2
37. Primahana, G.; Risdian, C.; Mozef, T.; Sudarman, E.; Köck, M.; Wink, J.; Stadler, M. Nonocarbolines A-E, β-Carboline Antibiotics Produced by the Rare Actinobacterium *Nonomuraea* sp. from Indonesia. *J. Antibiot.* 2020, 9(3), E126. https://doi.org/10.3390/antibiotics9030126
38. Primahana, G.; Risdian, C.; Mozef, T.; Wink, J.; Surup, F.; Stadler, M. Amycolatomycins A and B, Cyclic Hexapeptides Isolated from an *Amycolatopsis* sp. 195334CR. *J. Antibiot.* 2021, 10(3), 261. https://doi.org/10.3390/antibiotics10030261
39. Khodamoradi, S.; Hahnke, R.L.; Mast, Y.; Schumann, P.; Kämpfer, P.; Steinert, M.;Rückert, C.; Surup, F.; Rohde, M.; Wink, J. *Streptomonospora litoralis* sp. nov., a halophilic thiopeptides producer isolated from sand collected at Cuxhaven beach. *Antonie Van Leeuwenhoek*. 2021, 114(10): 1483-1496. https://doi.org/10.1007/s10482-021-01609-4
40. Khodamoradi, S.; Stadler, M.; Wink, J.; Surup, F. Litoralimycins A and B, New Cytotoxic Thiopeptides from *Streptomonospora* sp. M2. *Marin. Drugs.* 2020, 18(6), 280. https://doi.org/10.3390/md18060280
41. Liu, S.-W.; Jadambaa, N.; Nikandrova, A.A.; Osterman, I.A.; Sun, C.-H. Exploring the Diversity and Antibacterial Potentiality of Cultivable Actinobacteria from the Soil of the Saxaul Forest in Southern Gobi Desert in Mongolia. *Microorganisms*. 2022, 10(5), 989. https://doi.org/10.3390/microorganisms10050989
42. Su, Y.-S.; Wu, M.-D.; Chen, J.-J.; Cheng, M.-J.; Kuo, Y.-H.; Chai, C.-Y.; Kwan, A.-L. Secondary Metabolites with Anti-Inflammatory Activities from One Actinobacteria *Amycolatopsis taiwanensis*. *Molecules*. 2021,26(19), 5765. https://doi.org/10.3390/molecules26195765

43. Arn, F.; Frasson, D.; Kroslakova, I.; Rezzonico, F.; Pothier, J.F. Isolation and Identification of Actinomycetes Strains from Switzerland and their Biotechnological Potential. *Chimia (Aarau).* 2020. 74(5), 382-390. https://doi.org/10.2533/chimia.2020.382
44. Sharma, P.; Thakur, D. Antimicrobial biosynthetic potential and diversity of culturable soil actinobacteria from forest ecosystems of Northeast India. *Sci. Rep*. 2020, 10(1), 4104-4104. https://doi.org/10.1038/s41598-020-60968-6
45. Hu, D.; Lee, S. M.-Y.; Li, K.; Mok, K. M. Exploration of Secondary Metabolite Production Potential in Actinobacteria Isolated From Kandelia candel Mangrove Plant. *Front. Mar. Sci.* 2022, 9. https://doi.10.3389/fmars.2022.700685.
46. Hu, D.; Gao, C.; Sun, C.; Jin, T.; Fan, G.; Mok, K.M.; Lee, S.M. Genome-guided and mass spectrometry investigation of natural products produced by a potential new actinobacterial strain isolated from a mangrove ecosystem in Futian, Shenzhen, China. *Sci. Rep.* 2019, 9(1), 823. https://doi.org/10.1038/s41598-018-37475-w
47. Hu, D.; Sun, C.; Jin, T.; Fan, G.; Mok, K. M.; Li, K.; Lee, S. M.-Y. Exploring the Potential of Antibiotic Production From Rare Actinobacteria by Whole-Genome Sequencing and Guided MS/MS Analysis. *Front. Mar. Sci*, 2020, 11, 1540-1540. https://doi.org/10.3389/fmicb.2020.01540
48. Wang, D.; Wang, Y.; Ouyang, Y.; Fu, P.; Zhu, W. Cytotoxic p-Terphenyls from a Marine-Derived *Nocardiopsis* Species. *J. Nat. Prod.* 2019, 82(12), 3504-3508. https://doi.org/10.1021/acs.jnatprod.9b00963
49. Li, L.; Xu, Q.H.; Wang, X.T.; Lin, H.W.; Lu, Y.H. *Actinomadura craniellae* sp. nov., isolated from a marine sponge in the South China Sea. *Int. J. Syst. Evol. Microbiol*. 2019, 69(4), 1207-1212. https://doi.org/10.1099/ijsem.0.003295
50. Jiang, Z.-k.; Tuo, L.; Huang, D.-l.; Osterman, I. A.; Tyurin, A. P.; Liu, S.-w.; Lukyanov, D. A.; Sergiev, P. V.; Dontsova, O. A.; Korshun, V. A.; et al. Diversity, Novelty, and Antimicrobial Activity of Endophytic Actinobacteria From Mangrove Plants in Beilun Estuary National Nature Reserve of Guangxi, China. *Front. Microbiol*. 2018, 9, 868-868. https://doi.org/10.3389/fmicb.2018.00868
51. Sarveswari, H.B.; Kalimuthu, S.; Shanmugam, K.; Neelakantan, P.; Solomon, A.P. Exploration of Anti-infectives From Mangrove-Derived *Micromonospora sp*. RMA46 to Combat *Vibrio cholerae* Pathogenesis. *Front. Microbiol*. 2020, 11. https://doi.org/10.3389/fmicb.2020.01393
52. Indupalli, M.; Muvva, V.; Mangamuri, U.; Munaganti, R.K.; Naragani, K. Bioactive compounds from mangrove derived rare actinobacterium *Saccharomonospora oceani* VJDS-3. 3 Biotech. 2018a, 8(2), 103. https://doi.org/10.1007/s13205-018-1093-6
53. Siddharth, S.; Vittal, R.R.; Wink, J.; Steinert, M. Diversity and Bioactive Potential of Actinobacteria from Unexplored Regions of Western Ghats, India. *Microorganisms*. 2020, 8(2), E225. https://doi.org/10.3390/microorganisms8020225
54. Wang, R.-J.; Zhang, S.-Y.; Ye, Y.-H.; Yu, Z.; Qi, H.; Zhang, H.; Xue, Z.-L.; Wang, J.-D.; Wu, M. Three New Isoflavonoid Glycosides from the Mangrove-Derived Actinomycete *Micromonospora aurantiaca* 110B. *Mar. drugs*. 2019, 17(5), E294. https://doi.org/10.3390/md17050294
55. Marfil-Santana, M.D.; Martínez-Cárdenas, A.; Ruíz-Hernández, A.; Vidal-Torres, M.; Márquez-Velázquez, N.A.; Figueroa, M.; Prieto-Davó, A. A Meta-Omics Analysis Unveils the Shift in Microbial Community Structures and Metabolomics Profiles in Mangrove Sediments Treated with a Selective Actinobacterial Isolation Procedure. *Molecules*. 2021, 26(23), 7332. Retrieved https://doi.org/10.3390/molecules26237332
56. Lu, Q.-P.; Ye, J.-J.; Huang, Y.-M.; Liu, D.; Liu, L.-F.; Dong, K.; Razumova, E.A.; Osterman, I.A.; Sergiev, P.V.; Dontsova, O.A.; et al. Exploitation of Potentially New Antibiotics from Mangrove Actinobacteria in Maowei Sea by Combination of Multiple Discovery Strategies. *J. Antibiot.* 2019, 8(4), 236. https://doi.org/10.3390/antibiotics8040236.

57. Assad, B. M.; Savi, D. C.; Biscaia, S. M. P.; Mayrhofer, B. F.; Iantas, J.; Mews, M.; de Oliveira, J. C.; Trindade, E. S.; Glienke, C. Endophytic actinobacteria of *Hymenachne amplexicaulis* from the Brazilian Pantanal wetland produce compounds with antibacterial and antitumor activities. *Microbiol Res*. 2021, 248, 126768. https://doi.org/10.1016/j.micres.2021.126768.
58. Millán-Aguiñaga, N.; Soldatou, S.; Brozio, S.; Munnoch, J.; Howe, J.; Hoskisson, P.; Duncan, K. Awakening ancient polar Actinobacteria: diversity, evolution and specialized metabolite potential. *Microbiol*. 2019, 165. https://doi.org/10.1099/mic.0.000845
59. Silva, L. J.; Crevelin, E. J.; Souza, D. T.; Lacerda-Júnior, G. V.; de Oliveira, V. M.; Ruiz, A. L. T. G.; Rosa, L. H.; Moraes, L. A. B.; Melo, I. S. Actinobacteria from Antarctica as a source for anticancer discovery. *Sci. Rep*. 2020, 10(1), 13870. https://doi.org/10.1038/s41598-020-69786-2
60. Mehetre, G. T.; J, S. V.; Burkul, B. B.; Desai, D.; B, S.; Dharne, M. S.; Dastager, S. G. Bioactivities and molecular networking-based elucidation of metabolites of potent actinobacterial strains isolated from the Unkeshwar geothermal springs in India. *RSC Adv*. 2019, 9(17), 9850-9859. https://doi.org/10.1039/c8ra09449g
